# Supplementary material for: The characteristics of pre-existing humoral imprint determine efficacy of S. aureus vaccines and support alternative vaccine approaches
Source: Cell Rep Med. 2024 Jan 16;5(1):101360. doi: 10.1016/j.xcrm.2023.101360 (PMC10829788; doi:10.1016/j.xcrm.2023.101360)
Supplement: Document S1. Figures S1‒S6 [file mmc1.pdf]

Cell Reports Medicine, Volume *5*

Supplemental information

The characteristics of pre-existing humoral imprint determine efficacy of *S. aureus* vaccines and support alternative vaccine approaches

J.R. Caldera, Chih-Ming Tsai, Desmond Trieu, Cesia Gonzalez, Irshad A. Hajam, Xin Du, Brian Lin, and George Y. Liu

Cell Reports Medicine, Volume 5

Supplemental information

The characteristics of pre-existing humoral imprint determine efficacy of *S. aureus* vaccines and support alternative vaccine approaches

JR Caldera, Chih-Ming Tsai, Desmond Trieu, Cesia Gonzalez, Irshad A. Hajam, Xin Du, Brian Lin, George Y. Liu

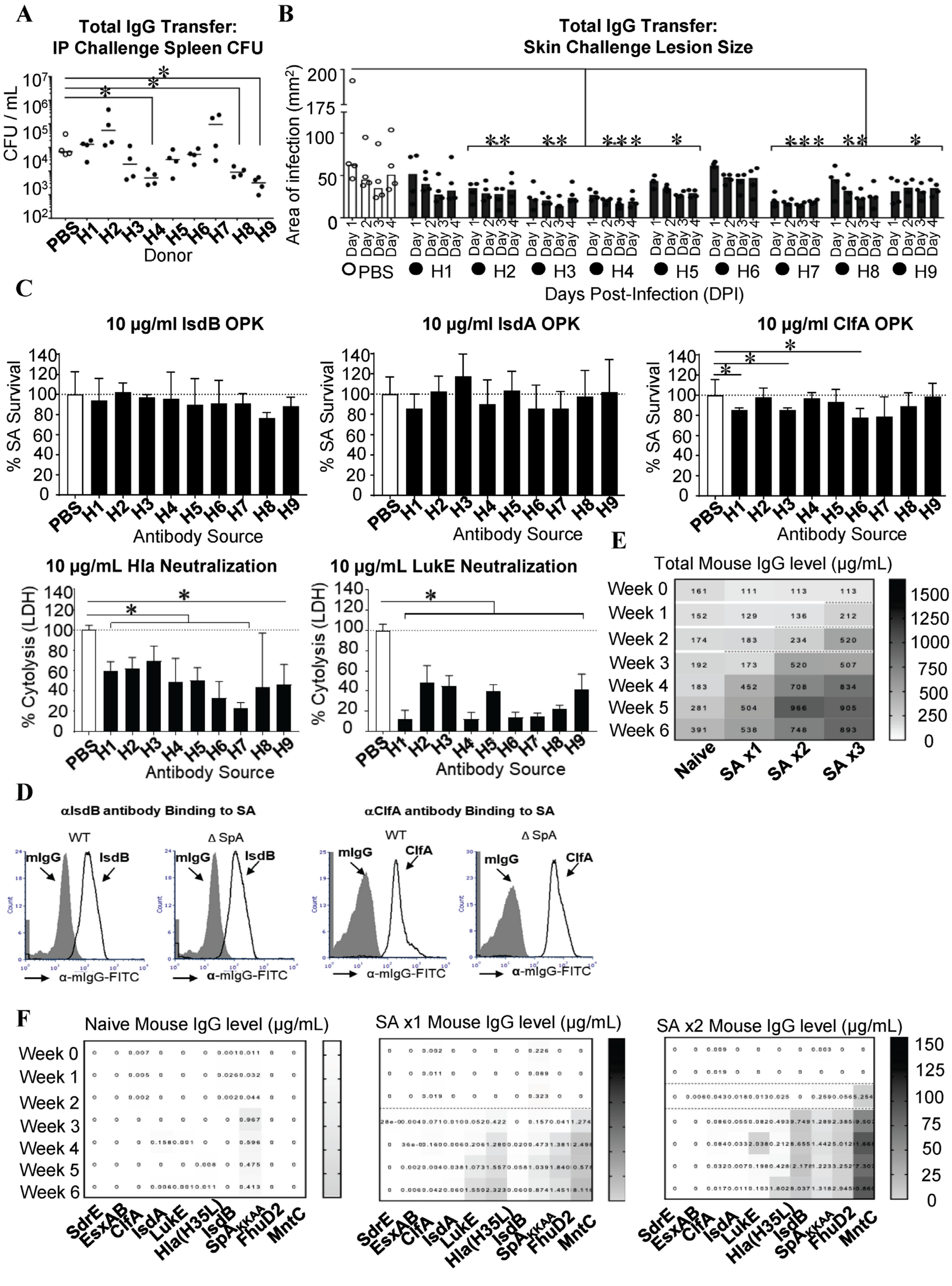
A
Total IgG Transfer: IP Challenge Spleen CFU
CFU / mL
Donor
PBS H1 H2 H3 H4 H5 H6 H7 H8 H9
B
Total IgG Transfer: Skin Challenge Lesion Size
Area of infection (mm2)
Days Post-Infection (DPI)
PBS H1 H2 H3 H4 H5 H6 H7 H8 H9
C
10 µg/ml IsdB OPK
10 µg/ml IsdA OPK
10 µg/ml ClfA OPK
% SA Survival
Antibody Source
10 µg/mL Hla Neutralization
10 µg/mL LukE Neutralization
% Cytolysis (LDH)
Antibody Source
D
αIsdB antibody Binding to SA
αClfA antibody Binding to SA
WT
Δ SpA
mIgG
IsdB
ClfA
α-mIgG-FITC
E
Total Mouse IgG level (µg/mL)
Week 0 161 111 113 113
Week 1 152 129 136 212
Week 2 174 183 234 520
Week 3 192 173 520 507
Week 4 183 452 708 834
Week 5 281 504 966 905
Week 6 391 538 748 893
Naive SA x1 SA x2 SA x3
1500 1250 1000 750 500 250 0
F
Naive Mouse IgG level (µg/mL)
SA x1 Mouse IgG level (µg/mL)
SA x2 Mouse IgG level (µg/mL)
Week 0 Week 1 Week 2 Week 3 Week 4 Week 5 Week 6
SdrE EsxAB ClfA IsdA LukE Hla(H35L) IsdB SpAKKAA FhuD2 MntC
150 125 100 75 50 25 0

Figure S1 Quantitative and functional assessments of human and mouse anti-SA humoral imprints, related to Figure 1.

(A) Post-challenge bacterial burden in kidneys of naïve C57BL/6 mice adoptively transferred total purified human IgG, or PBS, then challenged i.p. with SA.

(B) Skin lesion size after adoptive transfer of total human IgG or PBS, into naïve mice challenged i.d., measured daily for 4 days. Each point represents an individual mouse. Bar corresponds to the median.

(C) *In vitro* assessment of relative antigen-specific antibody function by OPK, and toxin-neutralization. Results are normalized to their respective control using normal mIgG.

(D) Validation of bacterial expression of IsdB/ClfA and binding of purified IsdB- andClfA- specific antibodies to log-phase growth of wild type SA LAC and isogenic SpA-mutant analyzed by flow cytometry. The gray histogram represents staining with FITC-conjugated anti-mouse IgG only; the bolded histogram line represents staining with IsdB antibodies, then secondary antibody.

(E) Total IgG in serum of naïve, 1x, and 2x SA-exposed mice, over 7 weeks. Dashed line denotes timepoints of SA exposure. Values in each cell correspond to the median titer from n=5 mice.

(F) Antigen-specific IgG corresponding to 10 proposed vaccine candidates, over 7 weeks from naïve and SA x1 and x2 infected mice. Dashed line denotes time of SA exposure. Values in each cell correspond to the median titer from n=5 mice. Ranking of antigens is based on average titer among the timepoints.

Unless otherwise stated, C57BL/6 mice were used. Each point represents an individual mouse (A and B); line or bar corresponds to the median (A and B). Bar represents group median; error bars represent means ± SD (C). n.s., not significant, $*p < 0.05$, $**p < 0.01$, and $***p < 0.001$. One-way ANOVA (A and C), Two-way ANOVA with Bonferroni correction (B).

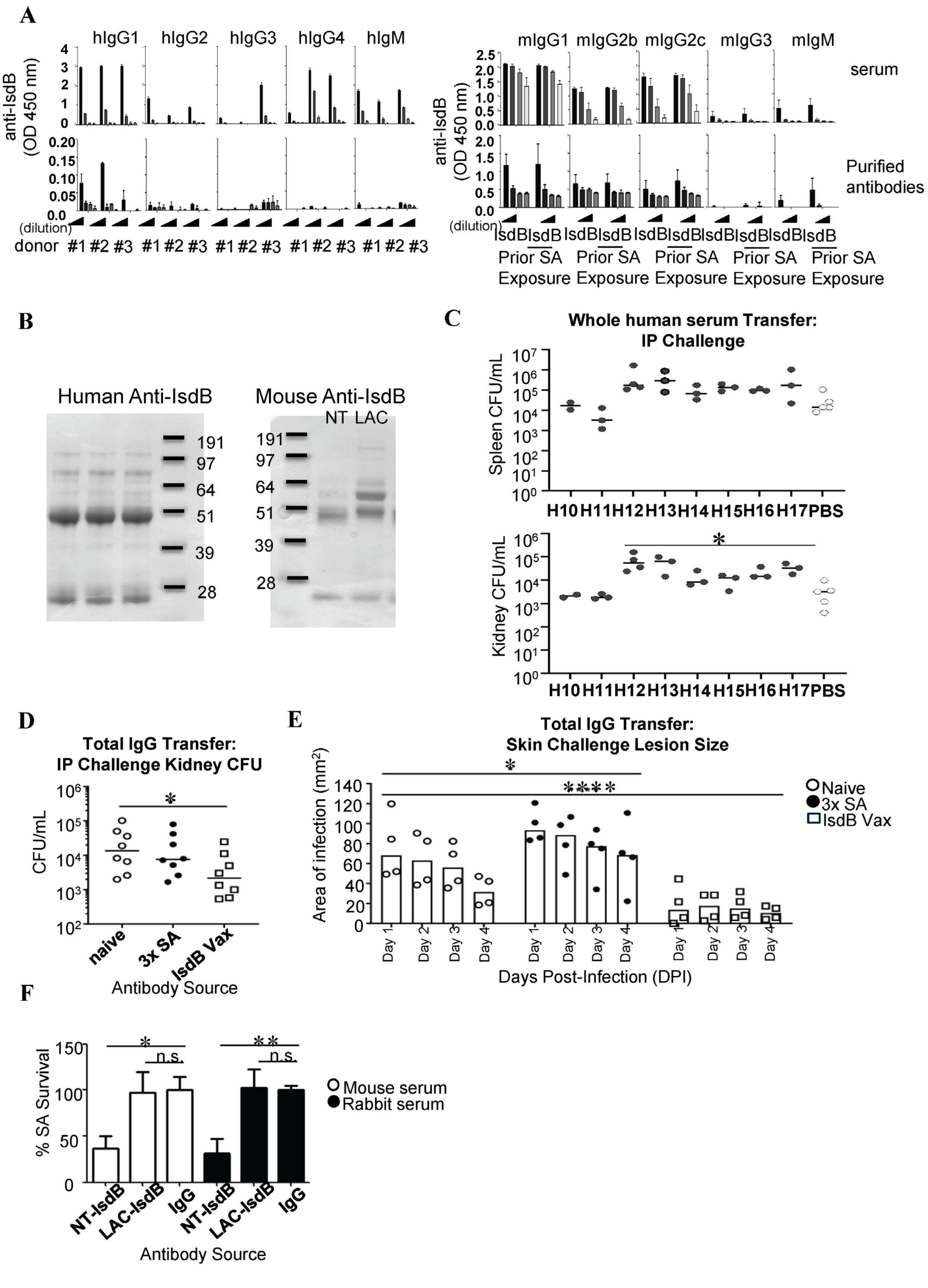
A
hIgG1
hIgG2
hIgG3
hIgG4
hIgM
anti-IsdB (OD 450 nm)
(dilution)
donor #1 #2 #3 #1 #2 #3 #1 #2 #3 #1 #2 #3 #1 #2 #3
mIgG1
mIgG2b
mIgG2c
mIgG3
mIgM
serum
Purified antibodies
IsdB
Prior SA Exposure
B
Human Anti-IsdB
Mouse Anti-IsdB
NT LAC
191
97
64
51
39
28
C
Whole human serum Transfer: IP Challenge
Spleen CFU/mL
Kidney CFU/mL
H10 H11 H12 H13 H14 H15 H16 H17 PBS
D
Total IgG Transfer: IP Challenge Kidney CFU
CFU/mL
naive
3x SA
IsdB Vax
Antibody Source
E
Total IgG Transfer: Skin Challenge Lesion Size
Area of infection (mm²)
Day 1
Day 2
Day 3
Day 4
Days Post-Infection (DPI)
Naive
3x SA
IsdB Vax
F
% SA Survival
n.s.
NT-IsdB
LAC-IsdB
IgG
Antibody Source
Mouse serum
Rabbit serum

Figure S2 Quantitative and functional assessments of human and mouse anti-SA humoral imprints, related to Figure 1.

(A) Isotype profiling of anti-IsdB antibodies from human (n=3) or mouse (n=5) whole or column-purified serum samples. IsdB-vaccinated naïve and SA-experienced mice were the sources of murine sera.

(B) SDS-PAGE analysis of representative purified antibodies: IsdB-specific antibodies purified from healthy human serum (n=3) and IsdB-vaccinated previously non-treated (NT) and IsdB-vaccinated SA-experienced mice (SA) (n=5). Please note that the 57KD band from the SA sample is 70% albumin,2% IgG and 9.93% IgM based on MS analysis.

(**C**) Post-i.p challenge SA burden in kidneys of mice adoptively transferred of 100μl human sera from subjects H10-H17 i.p.

(D) Post-challenge bacterial burden in kidneys of naïve C57BL/6 mice adoptively transferred total purified mouse IgG, then challenged i.p. with SA. Bar corresponds to the median.

(E) Skin lesion size after adoptive transfer of total mouse IgG, into naïve mice challenged i.d., measured daily for 4 days.

(F) *In vitro* comparison of relative protective serum anti-IsdB function by OPK with supplementation of mouse (left) or baby rabbit serum (right). Results are normalized to mIgG control.

Unless otherwise stated, C57BL/6 mice were used. Each point represents an individual mouse (C-E); line or bar corresponds to the median (C-E). Bar represents group median; error bars represent means ± SD (A and F). n.s., not significant, $*p < 0.05$, $**p < 0.01$, and $***p < 0.001$. One-way ANOVA (D and F), Two-way ANOVA with Bonferroni correction (E).

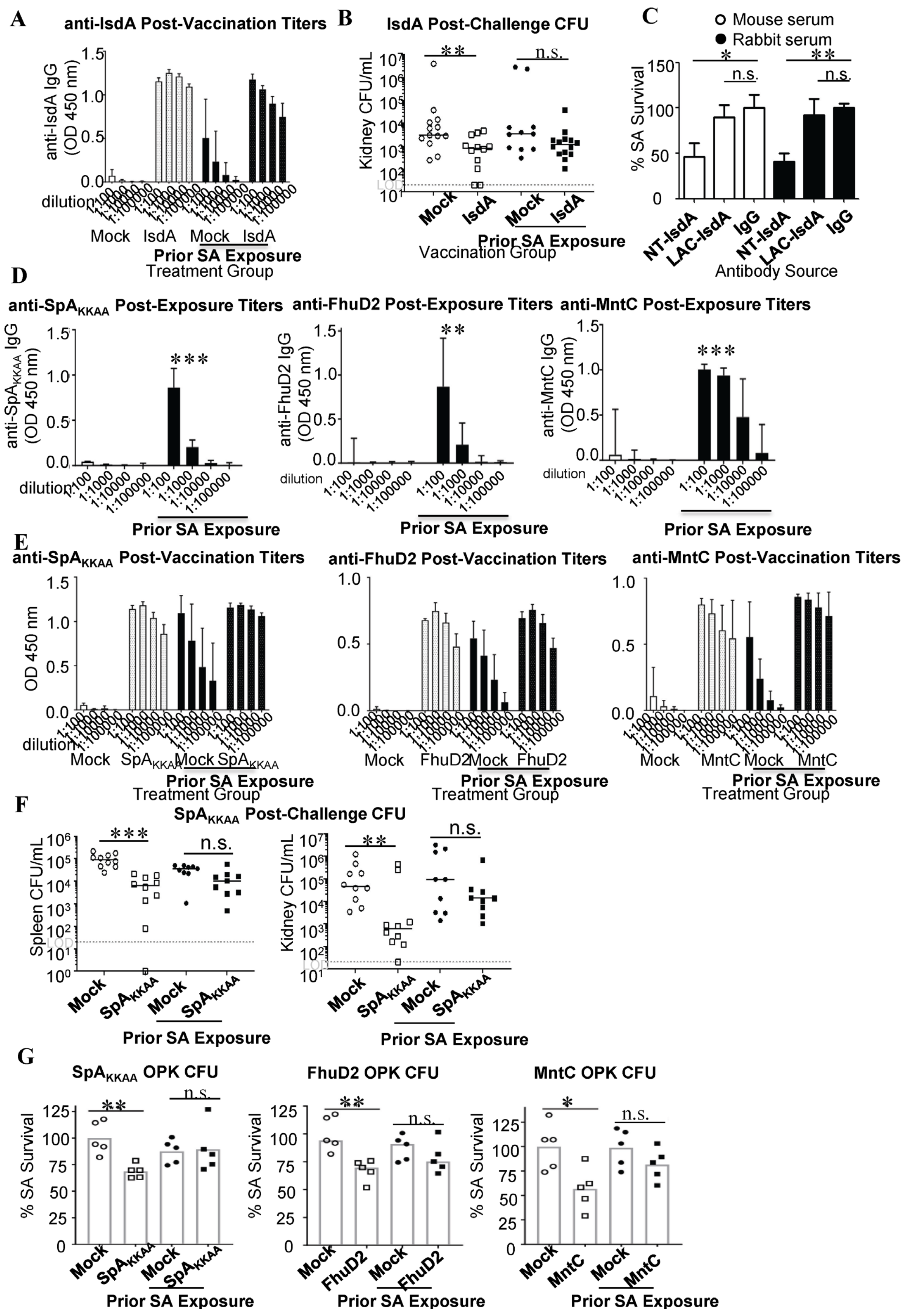
A
anti-IsdA Post-Vaccination Titers
anti-IsdA IgG (OD 450 nm)
dilution
1:100
1:1000
1:10000
1:100000
Mock
IsdA
Mock
IsdA
Prior SA Exposure
Treatment Group
B
IsdA Post-Challenge CFU
Kidney CFU/mL
LOD
**
n.s.
Mock
IsdA
Mock
IsdA
Prior SA Exposure
Vaccination Group
C
Mouse serum
Rabbit serum
% SA Survival
*
n.s.
**
n.s.
NT-IsdA
LAC-IsdA
IgG
NT-IsdA
LAC-IsdA
IgG
Antibody Source
D
anti-SpA$_{KKAA}$ Post-Exposure Titers
anti-FhuD2 Post-Exposure Titers
anti-MntC Post-Exposure Titers
anti-SpA$_{KKAA}$ IgG (OD 450 nm)
anti-FhuD2 IgG (OD 450 nm)
anti-MntC IgG (OD 450 nm)
***
**
***
dilution
Prior SA Exposure
E
anti-SpA$_{KKAA}$ Post-Vaccination Titers
anti-FhuD2 Post-Vaccination Titers
anti-MntC Post-Vaccination Titers
OD 450 nm
dilution
Mock
SpA$_{KKAA}$
Mock
SpA$_{KKAA}$
Mock
FhuD2
Mock
FhuD2
Mock
MntC
Mock
MntC
Prior SA Exposure
Treatment Group
F
SpA$_{KKAA}$ Post-Challenge CFU
Spleen CFU/mL
Kidney CFU/mL
LOD
***
n.s.
**
n.s.
Mock
SpA$_{KKAA}$
Mock
SpA$_{KKAA}$
Prior SA Exposure
G
SpA$_{KKAA}$ OPK CFU
FhuD2 OPK CFU
MntC OPK CFU
% SA Survival
**
n.s.
*
Mock
SpA$_{KKAA}$
FhuD2
MntC
Prior SA Exposure

Figure S3 Active immunizations targeting CWA are not protective in SA-experiencd mice, related to Figure 2.

(A) CWA-specific titers from n=5 vaccinated naïve and SA-experienced mice, 7 days after the last vaccination.

(B) Post-challenge bacterial burden in kidneys of mock- or IsdA-immunized naïve or SA-experienced mice.

(C) *In vitro* assessment of relative protective serum anti-IsdA function by OPK with serum supplement from mouse serum (open bar) or baby rabbit serum (black bar). Results are normalized to mIgG control.

(D-E) CWA-specific titers from n=10 naïve and SA-experienced mice, 7 days after the last SA exposure (D), and 7 days after last immunization (E).

(F) Post-challenge bacterial burden in kidneys of mock- or SpA_{KKAA}- immunized naïve or SA-experienced mice.

(G) *In vitro* assessment of relative protective serum function by OPK. Results are normalized to their respective control. Bar corresponds to the median. Each point represents an individual mouse. Bar corresponds to the median. Error bar corresponds to the range.

Unless otherwise stated, C57BL/6 mice were used. Bar represents group median; error bars represent means ± SD (A and C-E). Each point represents an individual mouse (B and F); bar corresponds to the median and dashed lines indicate the limit of detection (LOD) (B and F). $*p < 0.05$, $**p < 0.01$, and $***p < 0.001$. One-way ANOVA (B-C, and F-G).

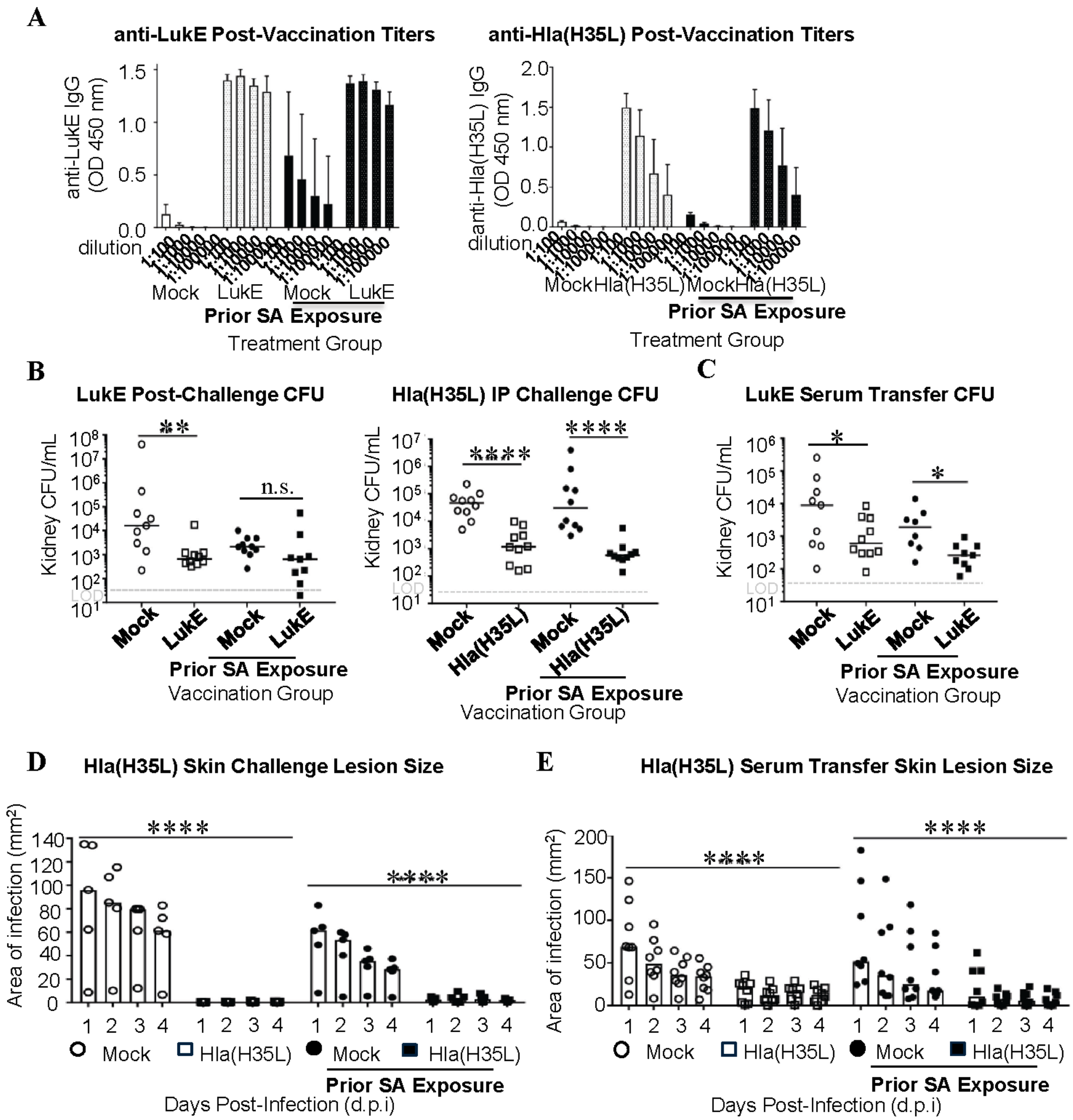

Figure S4 Active immunizations targeting toxin antigens are protective in SA-experienced mice, related to Figure 2.

(A) Toxin-specific titers from n=5 vaccinated naïve and SA-experienced mice, 7 days after the last vaccination. Bar corresponds to the median. Error bar corresponds to the range.

(B) Post-i.p. challenge bacterial burden in kidneys of mock-, LukE- or $Hla_{(H35L)}$-vaccinated naïve or SA-experienced mice. Each point represents an individual mouse. Line corresponds to the median.

(C) Post-i.p. challenge bacterial burden in kidneys of mice adoptively transferred serum from mock-, or LukE-vaccinated naïve or SA-experienced mice. Each point represents an individual mouse. Line corresponds to the median.

(D) Skin lesion size in mock- or $Hla_{(H35L)}$-vaccinated CD-1 naïve or SA-experienced mice measured daily for 4 days. Each point represents an individual mouse. Bar corresponds to the median. Dashed line corresponds to skin lesion size at 2d.p.i. of PBS control.

(E) Skin lesion size of CD-1 mice adoptively transferred serum from mock- or $Hla_{(H35L)}$-vaccinated mice measured daily for 4 days.

Unless otherwise stated, C57BL/6 mice were used. Bar represents group median. Each point represents an individual mouse (B-E); bar corresponds to the median and dashed lines indicate the limit of detection (LOD) (B and C). $*p < 0.05$, $**p < 0.01$, and $***p < 0.001$. One-way ANOVA (B and C) or two-way ANOVA with Bonferroni correction (D and E).

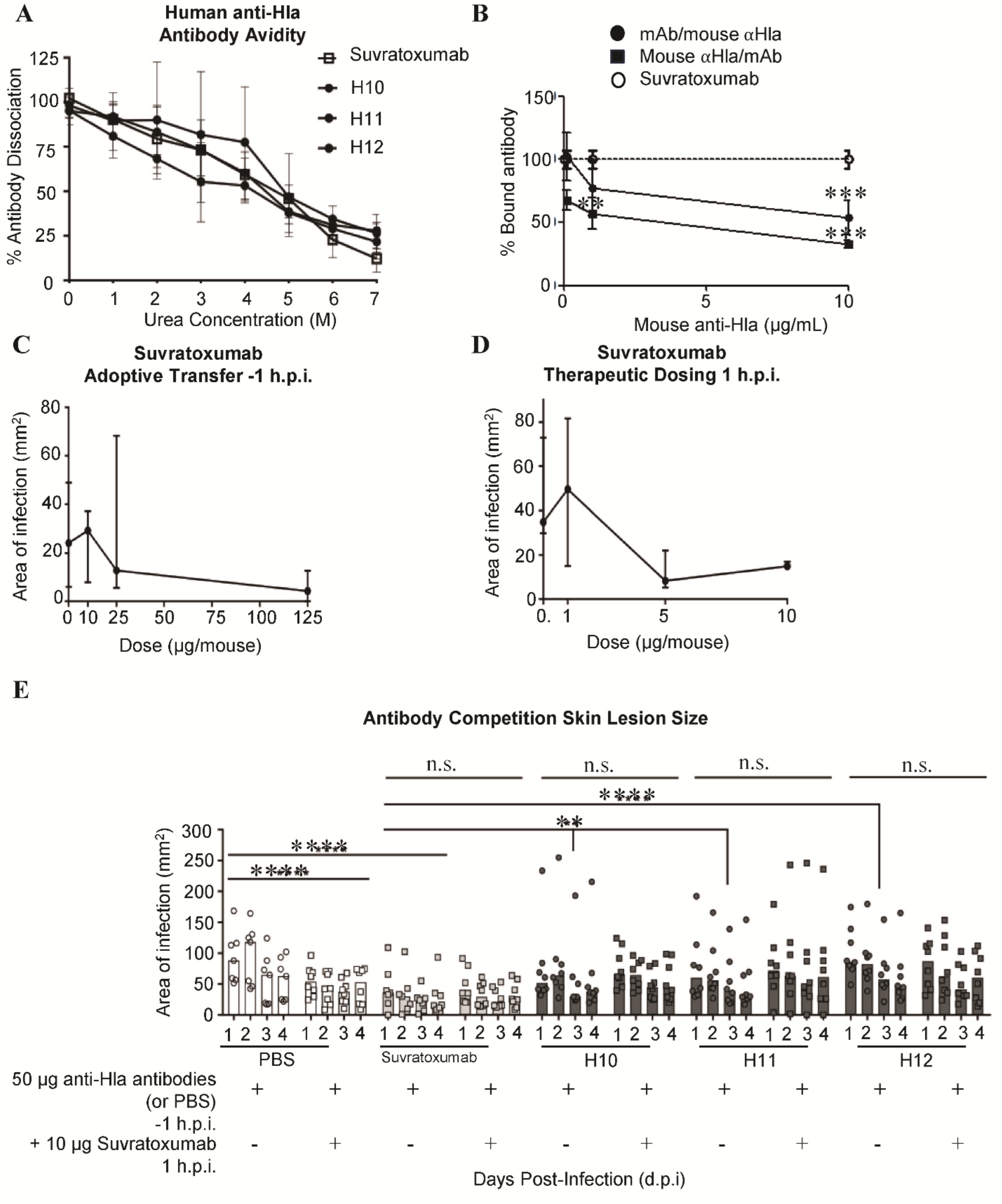

Figure S5 Pre-existing human anti-Hla antibodies blunt the efficacy of anti-Hla monoclonal antibody Suvratoxumab, related to Figure 3.

(A) Retention of anti-Hla antibodies (1µg/mL) with 0M-7M urea treatment. Each point represents 3 technical replicates. Error bar corresponds to range.

(B) Binding competition between purified mouse anti-Hla and Suvratoxumab, in an ELISA plate assay. Binding of recombinant Hla(H35L) by Suvratoxumab (1 ug/mL) or Hla-specific antibodies purified, from mice that have been SA-infected 3x (0.1-10 ug/ml). Suvratoxumab/mouse αHla: Suvratoxumab first, then mouse αHla; Mouse αHla/Suvratoxumab: mouse □Hla first, then Suvratoxumab; Suvratoxumab: Suvratoxumab only. Each point represents 3 technical replicates.

(C) Skin lesion size in CD-1 mice (n=3) that were adoptively transferred PBS, 10µg, 25µg, or 125µg Suvratoxumab, and then infected i.d. one hour later with SA. Skin lesions are measured 2 d.p.i.

(D) Skin lesion size of CD-1 mice after therapeutic administration of Suvratoxumab (10µg/mouse) or PBS 1h.p.i., measured daily for 4 days. Each point represents an individual mouse. Bar corresponds to the median. Dashed line corresponds to skin lesion size at 2d.p.i. of PBS control.

(E) Skin lesion size 1-4 d.p.i. in CD-1 mice passively immunized with anti-Hla antibodies (50µg/mouse), or PBS 1 hour prior to infection, followed by Suvratoxumab treatment (10µg/mouse) or PBS 1-hour post-infection. Each point represents an individual mouse.

Bar corresponds to the median. Error bars represent mean ± SD. $*p < 0.05$, $**p < 0.01$, and $***p < 0.001$. Student's t test (A, B) or two-way ANOVA with Bonferroni correction (E).

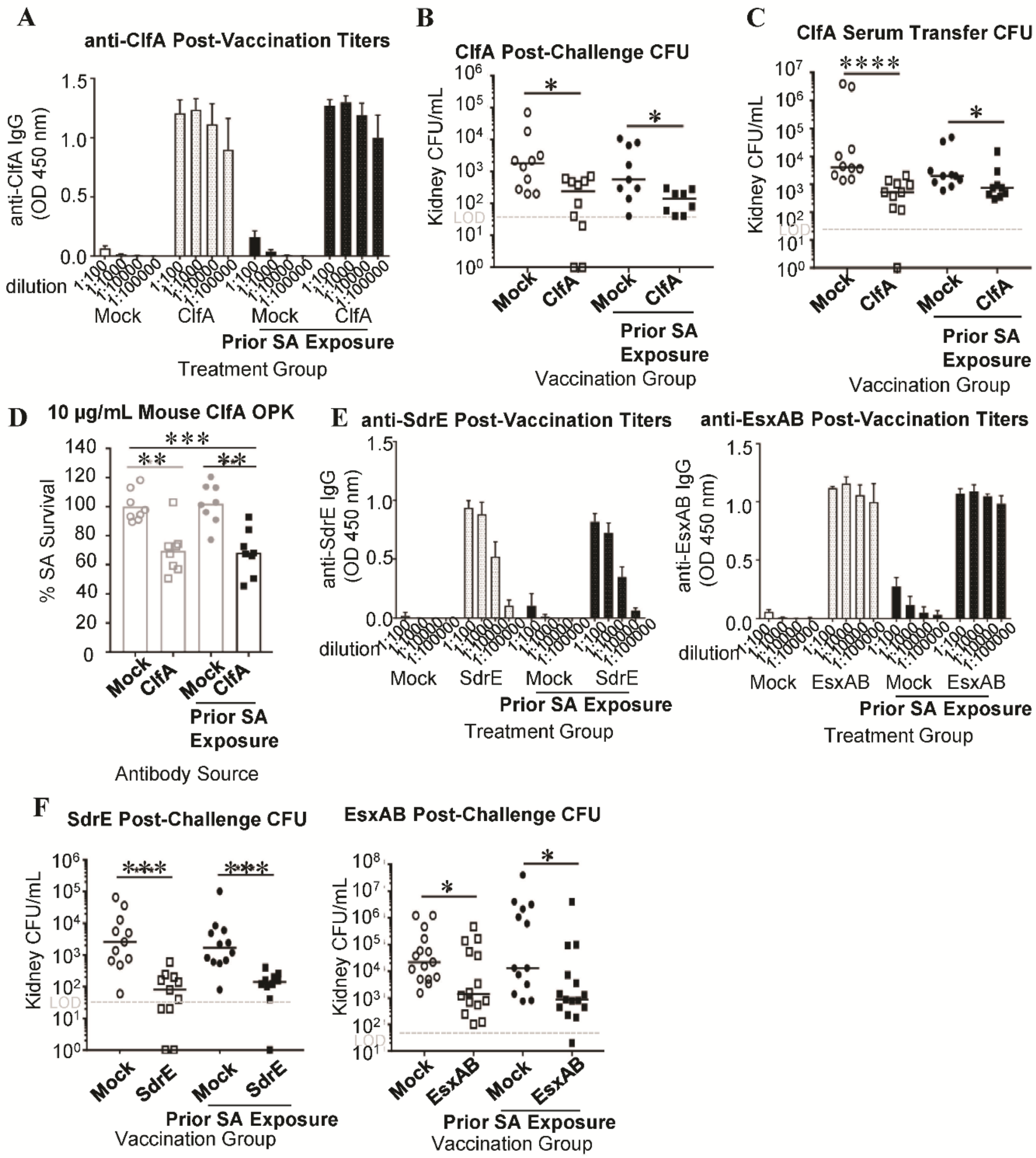

Figure S6 Subdominant SA CWA induce protective immunity in SA-experienced mice, related to Figure 4.

(A and E) ClfA-, SdrE- and EsxAB-specific titers from n=5 vaccinated naïve and SA-experienced mice, 7 days after the last SA vaccination. Bar corresponds to the median. Error bar corresponds to the range.

(B and F) Post-challenge bacterial burden in kidneys of mock- or (ClfA-, EsxAB- and SdrE-) vaccinated naïve or SA-experienced mice. Bar corresponds to the median.

(C) Post-challenge bacterial burden in kidneys of mice adoptively transferred serum from mock- or ClfA-vaccinated naïve or SA-experienced mice.

(D) *In vitro* assessment of relative ClfA-specific antibody function by OPK with purified anti-ClfA antibodies. Results are normalized to mIgG control. Bar corresponds to the mean of 8 biological *replicates from two independent experiments*.

C57BL/6 mice were used. Bar represents group median; error bars represent mean ± SD (A, E). Each point represents an individual mouse (B, C and F); bar corresponds to the median and dashed lines indicate the limit of detection (LOD) (B-C and F). ∗p < 0.05, ∗∗p < 0.01, and ∗∗∗p < 0.001.∗p < 0.05, ∗∗p < 0.01, and ∗∗∗p < 0.001. one-way ANOVA (B-D and F).
