# Supplementary material for: The characteristics of pre-existing humoral imprint determine efficacy of S. aureus vaccines and support alternative vaccine approaches
Source: Cell Rep Med. 2024 Jan 16;5(1):101360. doi: 10.1016/j.xcrm.2023.101360 (PMC10829788; doi:10.1016/j.xcrm.2023.101360)
Supplement: Document S2. Article plus supplemental information [file mmc2.pdf]

Cell Reports Medicine

Article

The characteristics of pre-existing humoral imprint determine efficacy of *S. aureus* vaccines and support alternative vaccine approaches

Graphical abstract

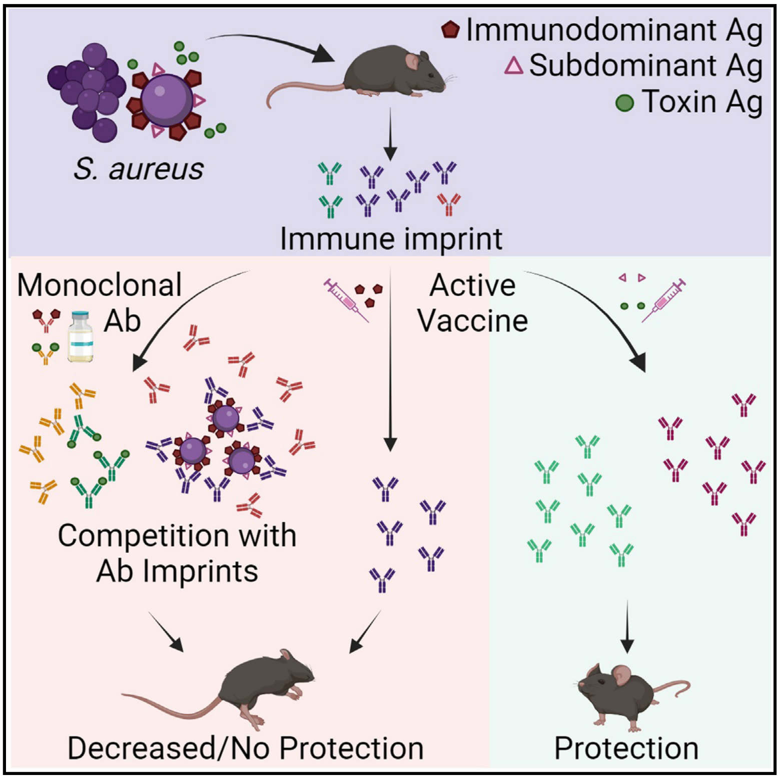

Highlights

- The target antigen and antibody titer predict outcome of *S. aureus* vaccines in mice
- Pre-existing human antibodies limit efficacy of anti-*S. aureus* monoclonal antibodies
- Interference from imprints is avoided by vaccinating against subdominant antigens

Authors

J.R. Caldera, Chih-Ming Tsai, Desmond Trieu, ..., Xin Du, Brian Lin, George Y. Liu

Correspondence

gyliu@ucsd.edu

In brief

There has been no successful vaccine against *S. aureus* after approximately 30 human trials. Humans are exposed to *S. aureus* early on in life. Caldera et al. show that the type of antigen target and the titer of pre-existing antibodies can predict the outcome of staphylococcal vaccines in pathogen-exposed mice.

Caldera et al., 2024, Cell Reports Medicine *5*, 101360
January 16, 2024 © 2023 The Authors.
https://doi.org/10.1016/j.xcrm.2023.101360

CellPress

Cell Reports Medicine

CellPress
OPEN ACCESS

Article

The characteristics of pre-existing humoral imprint determine efficacy of *S. aureus* vaccines and support alternative vaccine approaches

J.R. Caldera,[1,2,4] Chih-Ming Tsai,[2,4] Desmond Trieu,[2] Cesia Gonzalez,[2] Irshad A. Hajam,[2] Xin Du,[2] Brian Lin,[2] and George Y. Liu[2,3,5,*]
[1]Department of Biomedical Sciences, Cedars-Sinai Medical Center, Los Angeles, CA 90048, USA
[2]Department of Pediatrics, University of California, San Diego, La Jolla, CA 92093, USA
[3]Division of Infectious Diseases, Rady Children's Hospital, San Diego, CA 92123, USA
[4]These authors contributed equally
[5]Lead contact
*Correspondence: gyliu@ucsd.edu
https://doi.org/10.1016/j.xcrm.2023.101360

SUMMARY

The failure of the *Staphylococcus aureus* (SA) IsdB vaccine trial can be explained by the recall of non-protective immune imprints from prior SA exposure. Here, we investigate natural human SA humoral imprints to understand their broader impact on SA immunizations. We show that antibody responses against SA cell-wall-associated antigens (CWAs) are non-opsonic, while antibodies against SA toxins are neutralizing. Importantly, the protective characteristics of the antibody imprints accurately predict the failure of corresponding vaccines against CWAs and support vaccination against toxins. In passive immunization platforms, natural anti-SA human antibodies reduce the efficacy of the human monoclonal antibodies suvratoxumab and tefibazumab, consistent with the results of their respective clinical trials. Strikingly, in the absence of specific humoral memory responses, active immunizations are efficacious in both naive and SA-experienced mice. Overall, our study points to a practical and predictive approach to evaluate and develop SA vaccines based on pre-existing humoral imprint characteristics.

INTRODUCTION

As a successful human colonizer, *Staphylococcus aureus* (SA) balances a lifestyle of a symbiont and occasional deadly pathogen. Correspondingly, SA's role as a pathogen has earned it the categorical designation of "serious threat" to public health.[1] As bacteria that thrive in both community and healthcare settings, methicillin-resistant SA strains alone cause an estimated 323,000 hospitalizations, over 10,000 deaths, and an attributable financial cost of $1.7 billion annually.[1] Thus, developing an effective SA vaccine has been a major goal of US and global health organizations for the past decades. In pre-clinical settings, researchers have developed promising vaccines that have advanced to human clinical trials, yet none of the approximately 14 phase 2 and 3 trials have succeeded in meeting their respective target endpoints.[2,3] The reason behind vaccine efficacy differences between humans and animal has remained unclear.

One notable difference between humans and laboratory mice is their frequency of natural exposure to SA. Mice confined to laboratories rarely encounter human SA strains, whereas up to 50% humans are colonized or infected by 2 months of life and exhibit lifelong exposure, as evidenced by the increasing titers of anti-SA antibodies in normal human serum.[4–9] Furthermore, the robust titers of human anti-SA antibodies do not confer significant protection against SA infections.[10,11] This prompted us to speculate that SA vaccines might have failed because they recalled non-protective pre-existing humoral responses, borrowing from the "original antigenic sin" hypothesis that was used to explain persistently poor influenza vaccine efficacy across seasons.[12]

We previously provided support for the hypothesis by recapitulating the failure of the human clinical trial vaccine targeting SA iron-regulated surface determinant (Isd) B protein in a mouse model of prior SA exposure.[13] We showed that primary SA infection in mice induced IsdB-specific memory responses that were not protective. Subsequent IsdB immunization of SA-experienced mice preferentially recalled the non-protective humoral imprint leading to an ineffective vaccine response. Additionally, we showed that any potentially protective effect of anti-IsdB antibodies generated by vaccination was blunted by direct competition against the non-protective antibodies.[13]

Having established proof of principle that humoral imprint to IsdB adversely impacted vaccine responses, we next aimed to determine if immune imprinting could also explain other SA vaccine trial failures. Early anti-SA vaccine approaches targeted cell-wall-associated antigens (CWAs) that induce opsonophagocytic killing of SA. Extensive research identified a long list of promising targets including the ion transporter IsdA, manganese

This is an open access article under the CC BY license (http://creativecommons.org/licenses/by/4.0/).

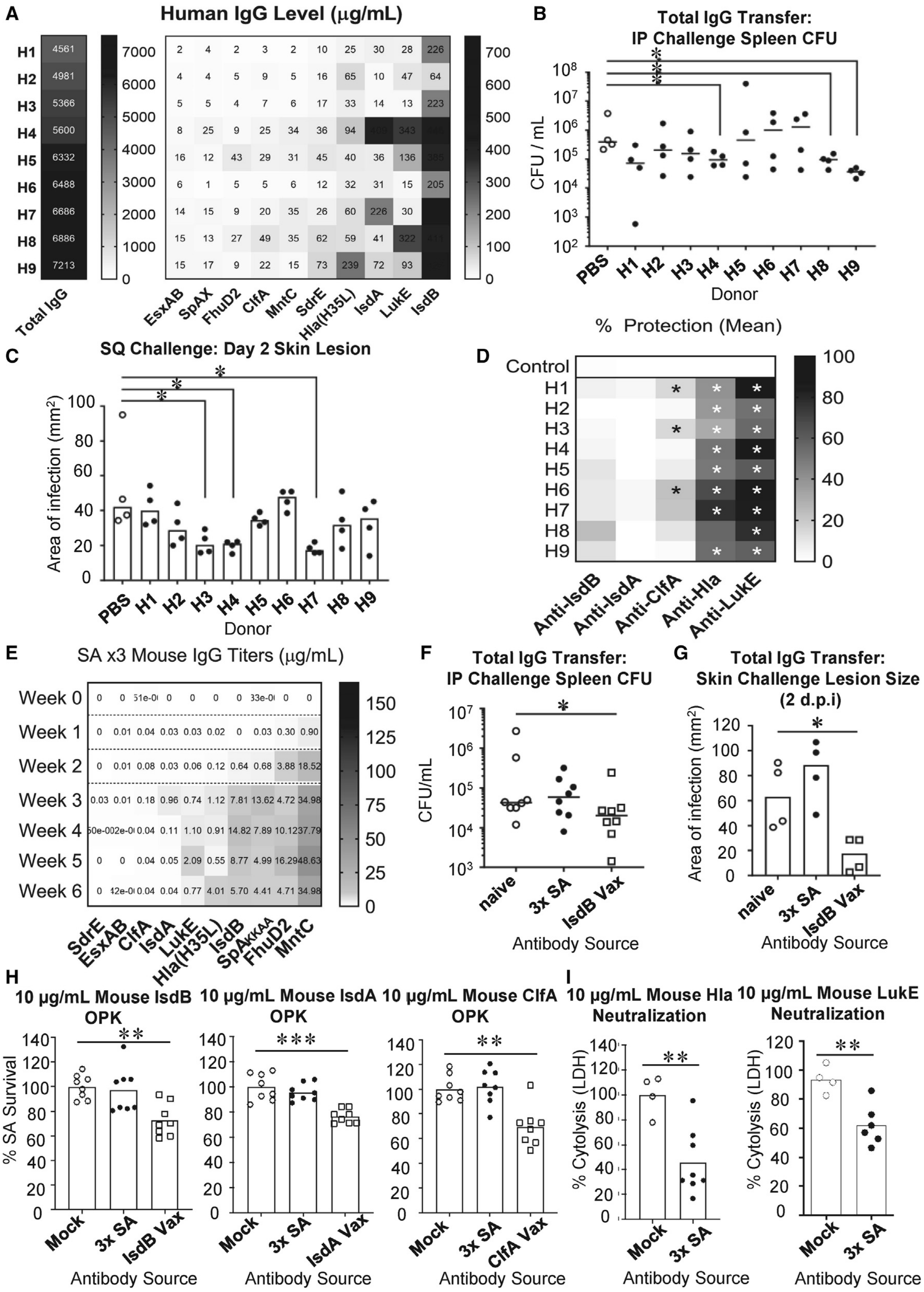

(legend on next page)

transporter C (MntC), and ferric hydroxymate uptake protein D2 (FhuD2) and the staphylococcal virulence factors staphylococcal protein A (SpA), clumping factor A (ClfA), serine-aspartate repeat protein E (SdrE), and Ess-secreted proteins A,B (EsxA/B) in naive rodents.[14–22] Vaccination against CWAs, however, have proven to be unsuccessful to date, whether evaluated for the prevention or treatment of SA infections.[2,23–26] As a result, more recent SA vaccine trials have shifted to the neutralization of staphylococcal cytolytic toxins, such as staphylococcal leukocidin E (LukE), and the extensively characterized alpha hemolysin (Hla) as a way to minimize damage to the host rather than primarily reduce bacterial burden.[17,22,27–29] Remarkably, both active and passive platforms of immunization against SA toxins were also met with failures.[30]

Given this background, we sought to understand key properties of the human anti-staphylococcal immunome with the aim to determine if and how certain characteristics of the SA humoral imprints, or lack thereof, affect subsequent vaccination.

RESULTS

Characterization of vaccine-relevant SA humoral imprints suggests classes of antigens with distinct antibody protection profiles

To profile the naturally circulating human anti-SA antibody immunome, we studied the humoral responses against 10 SA antigens that represent major CWAs and toxin vaccine antigens, including several targets of failed clinical trials.[15–20,31] We set out to profile the total and antigen-specific humoral imprints against these select antigens, focusing on two antibody traits that appeared sufficient to predict IsdB vaccine failure in our previous study: immunoglobulin G (IgG) titer and specific protective efficacy.

We collected sera from 9 healthy adult volunteers and quantified total and SA-antigen-specific IgG, ordered from low to high in Figure 1A. All subjects demonstrated circulating serum anti-SA antibodies, and the relative abundance of each antigen-specific antibody was similar across subjects (Figure 1A). Specifically, antibodies against IsdB were consistently the highest among the donors, while antibodies against EsxAB were consistently the lowest.

To first determine the overall protective function of these preexisting antibodies, we purified total human IgG from 700 μL human serum. We adoptively transferred the IgG into naive mice by intravenous (i.v.) injection and then challenged the recipients systemically (intraperitoneal [i.p.]) with 3.5×10^7 colony-forming unit (CFU) SA, or locally (intradermal [i.d.]) with 5×10^7 CFU SA, 24 h later. In both models of infection, transferred antibodies from only one-third of the human donors were protective against SA infection (Figures 1B, 1C, S1A, and S1B), and the antibody samples that conferred protection in the soft tissue and systemic infection models overlapped but were not identical. However, if i.d. infection protection was measured over 4 days, 75% donor antibodies were now protective and overlapped fully with the samples that were protective against systemic infection (Figure S1B). Overall, our data suggest that human humoral responses confer modest to moderate levels of protection against SA.

To determine the protective capacity of antibodies against specific staphylococcal CWAs and toxins, we focused on five prominent vaccine candidates that included four antigens that had been targets in clinical trials (IsdB, IsdA, ClfA, Hla, and LukE).[25,30,32,33] We performed opsonophagocytic killing (OPK) and toxin neutralization assays based on prior suggestion that these assays correlated most closely with protective function of antibodies against CWAs and toxins.[34] We purified antibodies to the CWAs IsdB, IsdA, and ClfA for OPK and the toxins LukE and Hla for neutralization assay. Human anti-IsdB, -IsdA, and -ClfA antibodies were relatively ineffective in OPK of SA (Figures 1D and S1C), which was not the result of low antigen expression on SA cell surface (Figure S1D). The finding was compatible with the lack of

Figure 1. Quantitative and functional assessments of human and mouse anti-SA humoral imprints
(A) Total and antigen-specific IgG corresponding to 10 proposed vaccine candidates from 9 healthy human donors. Ranking of human donors is based on total IgG per donor. Ranking of antigens is based on total quantity per antigen among all donors from lowest to highest. Value in each cell corresponds to titer in μg/mL serum.
(B) Post-challenge bacterial burden in spleen of naive C57BL/6 mice adoptively transferred 25 μg total purified human IgG or PBS and then challenged i.p. with SA.
(C) Skin lesion size 2 days post-infection (dpi) in CD1 mice adoptively transferred 25 μg total purified human IgG or PBS and then challenged i.d. with SA.
(B and C) Each point represents an individual mouse. Line or bar corresponds to the median.
(D) *In vitro* assessment of relative antigen-specific antibody function by OPK (anti-IsdB, anti-IsdA, anti-ClfA) and toxin neutralization (anti-Hla and anti-LukE). 10 μg/mL purified antibodies were used in OPK. Results are normalized to their respective control using normal mouse IgG (mIgG). The color of each cell corresponds to the mean of 8 technical replicates from two independent experiments. Statistically significant p values are noted within the cell. OPK made use of murine bone-marrow-derived neutrophils.
(E) Mouse antigen-specific IgG corresponding to 10 proposed vaccine candidates over 7 weeks. Dashed line denotes time of SA exposure. Values in each cell correspond to the median level from n = 5 mice. Ranking of antigens is based on average titer among the time points from lowest to highest.
(F) Post-challenge bacterial burden in spleen of naive C57BL/6 mice adoptively transferred 25 μg total purified mIgG from SA-infected or IsdB-vaccinated donor mice. The mice were subsequently challenged i.p. with SA.
(G) Skin lesion size 2 dpi in CD1 mice adoptively transferred 25 μg total purified mIgG from SA-infected or IsdB-vaccinated donor mice. The mice were subsequently challenged i.d. with SA.
(F and G) Each point represents an individual mouse. Line or bar corresponds to the median.
(H and I) *In vitro* assessment of relative antigen-specific antibody function by OPK (anti-IsdB, IsdA, and ClfA) (H) or toxin neutralization (anti-Hla and LukE) (I) with antibodies purified from 3× SA or naive vaccination. 10 μg/mL purified antibodies were used in OPK or toxin-neutralization assay. Results are normalized to the respective mIgG control. OPK made use of murine bone-marrow-derived neutrophils. Unless otherwise stated, C57BL/6 mice were used.
Each data point represents an individual mouse (B, C, F, and G); bar corresponds to the mean (B and C). Bar corresponds to the mean of 8 technical replicates from 2 independent experiments (H and I). n.s., not significant, *$p < 0.05$, **$p < 0.01$, and ***$p < 0.001$; Student's t test (I) or one-way ANOVA (A–H).

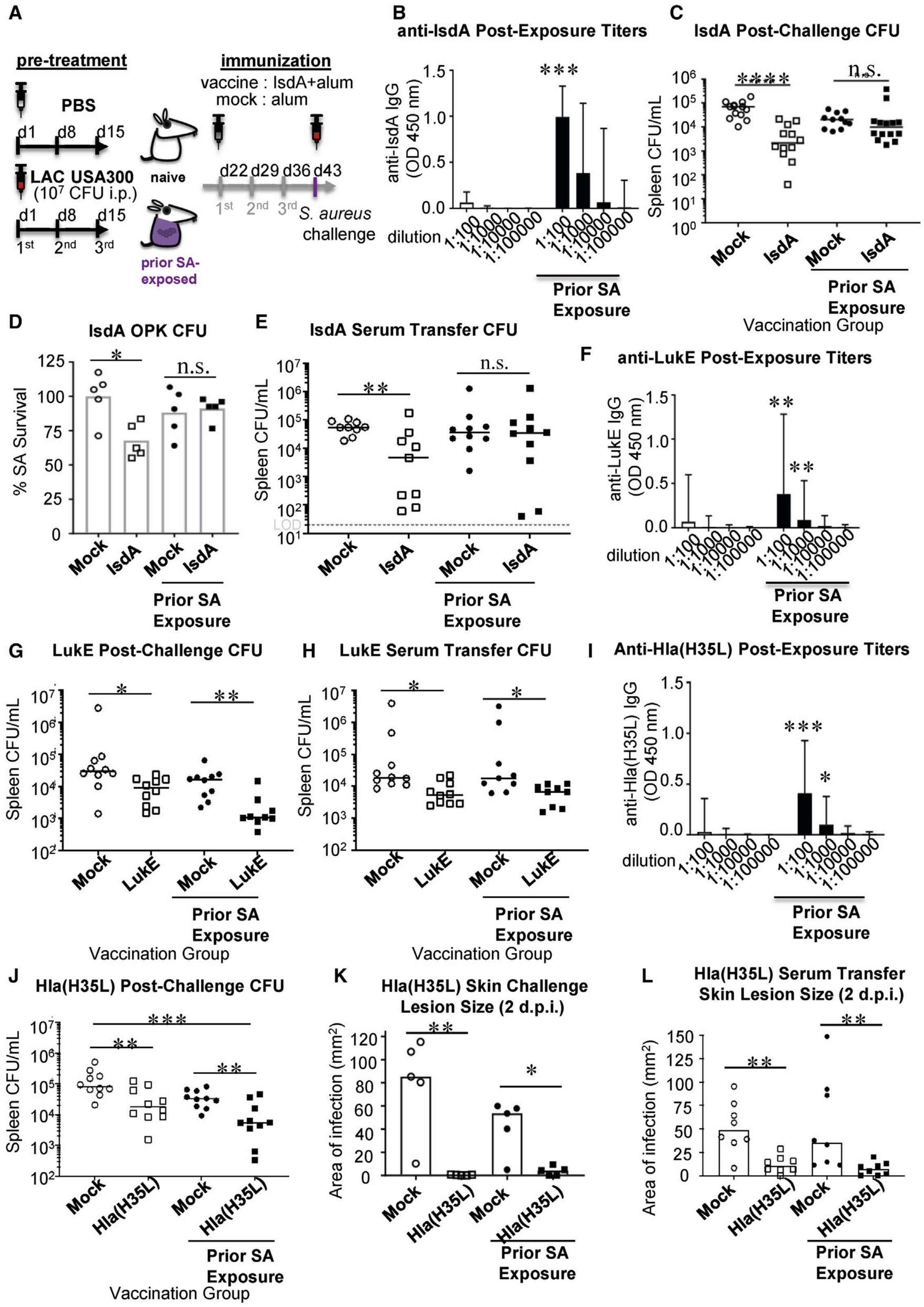

(legend on next page)

protective efficacy of human IsdB antibodies that was described in our previous publication.[13] In comparison, anti-LukE and -Hla antibodies from most subjects showed robust neutralizing activity (Figures 1D and S1C).

To explore the translation of these observations to laboratory animal models, we utilized our previously described model of vaccination in which mice are serially exposed to SA using self-limiting infectious doses. As before, we analyzed IgG antibody titers and protective antibody functions by analyzing sera from naive mice and mice previously infected with SA using protective serum from naive-vaccinated mice as positive control. Total IgG increased with frequency of SA exposure and with time from the last SA exposure (Figures 1E, S1E, and S1F). As with human sera, exposure of mice to SA induced titers of anti-SA antibodies that varied between antigens and frequency of exposures, with 3 SA infections demonstrating the most stable antibody levels (Figure 1E).

In Ig subclass profiling of (representative) whole-serum and purified anti-IsdB human and mouse antibodies, we noted that some human IgG3, IgG4, and IgM were excluded during purification on protein A (Figures S2A and S2B). In comparison, mouse IgM was co-purified with protein G. Hence, we further tested if, without purification, human sera would have been protective. Adoptive transfer of whole human sera failed to protect mice from SA challenge (Figure S2C). We conclude that human and mouse anti-SA antibodies are modestly or non-protective at the concentrations tested.

To compare the immunogenicity of SA antigens in humans and mice, we investigated the ranking titer of mouse and human SA-specific antibodies based on their abundance from low to high (Figures 1A and 1E). Overall, there was no clear correlation between the order of SA antibody titers in C57BL/6 mice and humans. While some antigens consistently appear to be highly (IsdB) or minimally (EsxAB) immunogenic in both species, others (MntC) demonstrated contrasting levels and were highly immunogenic in C57BL/6 mice but only modestly immunogenic in humans.

In functional assays, total IgG from mice infected 3× with SA were not protective against either systemic (i.p.) or soft tissue (i.d.) SA challenge compared to control antibodies derived from (IsdB-)vaccinated naive mice (Figures 1F, 1G, S2D, and S2E). Consistent with the human data, anti-IsdB, -IsdA, and -ClfA antibodies from infection did not mediate OPK compared to their respective vaccine-derived controls, while infection-induced anti-Hla and -LukE antibodies were neutralizing (Figures 1H, 1I, and S2F).

Overall, these findings suggest that both human and murine anti-CWA antibodies are non-opsonophagocytic and that anti-SA toxin antibodies are neutralizing. Our data also point to the utility of SA-exposed mouse for modeling of human vaccination.

Efficacy of active vaccination in the setting of protective imprints against SA toxins

Prior studies have correlated anti-Hla and LukE antibodies with improved clinical outcomes in cases of SA sepsis and soft-tissue infections, respectively.[35–37] Consistent with these studies, we showed that anti-toxin antibodies induced by SA are neutralizing, in stark contrast to anti-CWA antibodies, which are non-opsonophagocytic (Figure 1). Therefore, we ask if protective imprints, in contrast to non-protective imprints, would lead to different vaccination outcome in mice previously exposed to SA.

To begin, we sought to corroborate the broader suppressive effect of non-protective humoral imprint on SA vaccination in a murine model that we established previously with IsdB vaccination (Figure 2A). We focused on SA antigens that induced moderate or high titers of antibody imprints after SA infection, which we define to have median titers greater than the lowest quartile (Q1: 0.322 μg/mL) after 3× SA exposure. SA exposure in humans or mice induces robust levels of antibodies to surface-associated protein IsdA (Figures 1A, 2B, and S3A) that, like IsdB, facilitates transport of heme into SA, although it has no significant sequence homology to IsdB. As with IsdB, human and mouse anti-IsdA antibodies were poorly opsonophagocytic (Figures 1D, 1H, 2D, and S3C), and IsdA vaccination was protective in naive mice but not in mice previously exposed to SA (Figures 2C and S3B). Adoptive transfer of sera corroborated the loss of vaccine humoral protection in SA pre-exposed mice (Figure 2E). In addition to IsdB and IsdA vaccination, vaccination against three other moderate-to-high-titer antigens, SpA_{KKAA}, FhuD 2, and MntC (Figure S3D), also produced non-opsonophagocytic sera (Figures S3E and S3G) and were non-protective in SA-infected mice (*in vivo* Fhud2 and MntC data were previously reported,[13] and SpA_{KKAA} vaccine findings are shown in Figure S3F). Altogether, these

Figure 2. Active immunizations targeting toxin antigens are protective in SA-experienced mice

(A) Schematic of experimental design: naive mice are injected i.p. with PBS or 2.5 × 10^7 CFU SA once every 7 days × 3 weeks. Seven days following the last injection, SA-infected (or PBS-injected) mice are immunized with Alum or Alum/IsdA i.p. weekly × 3. Seven days after the last immunization, the mice are challenged with 2.5 × 10^7 CFU SA. Bacterial burden in spleen and kidneys are enumerated after 24 h.

(B) IsdA-specific titers from n = 10 naive and SA-experienced mice 7 days after the last SA exposure.

(C) Post-challenge bacterial burden in spleen of mock- or IsdA-immunized naive or SA-experienced mice.

(D) *In vitro* assessment of relative protective serum function by OPK. 10 μg/mL purified antibodies were used in OPK. Results are normalized to mIgG control. Bar corresponds to the median. Each point represents serum from an individual mouse.

(E) Post-challenge bacterial burden in spleen of naive C57BL/6 mice adoptively transferred 100 μL serum from mock- or IsdA-immunized naive or SA-experienced mice.

(F and I) LukE and Hla-specific titers from n = 10 naive and SA-experienced mice 7 days after last SA exposure.

(G and J) Post-i.p. challenge bacterial burden in mock- or (LukE- or $Hla_{(H35L)}$-)vaccinated naive or SA-experienced mice.

(H) Post-challenge bacterial burden in spleen of naive mice adoptively transferred serum from mock- or LukE-vaccinated mice and then challenged i.p. with SA.

(K) Skin lesion size 2 dpi in mock- or Hla-vaccinated naive or SA-infected mice challenged i.d. with SA.

(L) Skin lesion size 2 dpi in mice adoptively transferred serum from mock- or $Hla_{(H35L)}$-vaccinated mice. Unless otherwise stated, C57BL/6 mice were used.

Bar represents group median, and error bars represent means ± SD (B, F, and I). Each point represents an individual mouse (C, E, G, H, and J–L); line or bar corresponds to median (C, E, G, H, and J–L). Bar corresponds to the median (D). n.s., not significant, *p < 0.05, **p < 0.01, and ***p < 0.001; Student's t test (B, F, and I) or one-way ANOVA (C–E, G, H, and J–L).

data strongly suggest that moderate-to-high levels of non-protective imprints predict vaccine failure.

Next, to address the effect of protective antibody imprints on subsequent active vaccinations, we investigated two SA toxin candidates that induce neutralizing antibodies after SA exposure (Figures 1D and 1I). We immunized naive or SA-exposed mice with either the LukE subunit of the LukED toxin complex or detoxified Hla(H35L) toxoid.[38] SA infection induced moderate to high levels of LukE- and Hla-specific antibody titers (Figures 2F and 2I). LukE or Hla vaccination induced serum-transferable protection in both naive and SA-experienced mice (Figures 2G, 2H, 2J, and S4A–S4C). Since Hla is a major virulence factor in staphylococcal soft-tissue infections, we also investigated and determined that humoral response to Hla vaccination is protective in soft-tissue infection in both naive and SA-exposed mice (Figures 2K, 2L, S4D, and S4E).

Overall, these data strongly suggest that protective humoral imprints have no significant effect on active SA vaccination, whereas non-protective imprint negatively impacts active SA vaccination. Because SA toxins induce neutralizing protective antibodies, anti-toxin vaccines could represent an effective means of generating protective immunity, whether by expanding protective imprints or by generating *de novo* protective antibodies.

Human anti-Hla humoral imprints reduce the efficacy of anti-Hla passive immunization

To date, most SA vaccine trials have consisted of passive immunizations. Considering the protective function of natural anti-toxin human antibodies,[3] many investigators have sought to capitalize on the potential of anti-toxin monoclonal antibodies to ameliorate human SA infections.

Among the monoclonal antibody trials is a study that explored the efficacy of the anti-Hla monoclonal antibody suvratoxumab in the treatment of SA pneumonia, where the pathogenic role of Hla is well established.[30] Published data suggest that human infection-generated anti-Hla antibodies correlate with protection.[3,39,40] Thus, it was anticipated that treatment with suvratoxumab, a fully humanized antibody with an extended serum half-life, would be effective. Unfortunately, the findings of the SAATELLITE phase 2 pilot trial showed that the incidence of SA infection between treatment and placebo recipients was not significantly different.[30]

Our study of the IsdB vaccine posits that interference occurs not only by the preferential cellular recall of prior imprint but also by the ability of the amplified non-protective antibody imprint to directly blunt *de novo* vaccine-derived protection. Hence, we hypothesized that SA-induced antibodies, if present in sufficient amount, could interfere with passively administered therapeutic antibodies to reduce their efficacy.

In vitro, we showed that natural human anti-Hla antibodies neutralized Hla cytotoxic activity but were 10-fold less effective than suvratoxumab (Figure 3A). Using an avidity assay where target-bound antibodies were treated with increasing concentrations of a chaotropic agent, urea, that disrupts binding of antibodies, we showed that suvratoxumab bound to Hla with similar avidity to natural human anti-Hla antibodies (Figures 3B and S5A). To determine how effectively suvratoxumab competed against natural human anti-Hla antibodies, we incubated either antibody with recombinant Hla bound on a plate and then added the competing antibody. Purified human anti-Hla antibodies resisted displacement by the added suvratoxumab and successfully displaced pre-bound suvratoxumab (Figure 3C). In a similar competition assay, murine anti-Hla antibodies also displayed suvratoxumab when bound first to Hla on the ELISA plate, although suvratoxumab can also displace the murine antibodies (Figure S5B).

To assess the effect of human Hla antibodies on suvratoxumab protection against SA skin and soft-tissue infection, we first determined the dose of suvratoxumab that reduced skin lesion size by $\geq$50% when administered 1 h prior to (50 μg/mouse) or 1 h after (10 μg/mouse) SA infection (Figures S3C and S5D). Then, we administered to naive mice 50 μg/mouse human anti-Hla antibodies or PBS, infected the mice i.d. with SA 1 h after, and then administered a therapeutic dose of suvratoxumab (10μg/mouse) 1 h post-infection. As shown in Figures 3D and S5E, natural human anti-Hla antibodies were less effective than suvratoxumab in reducing SA skin lesions (Figures 3D and S5E). However, suvratoxumab had no therapeutic efficacy in mice pre-infused with purified human anti-Hla antibodies (Figures 3D and S5E). Although this study is not intended to mimic the conditions and dosing of monoclonal antibodies used in the clinical trial, it demonstrates that natural anti-Hla antibodies could suppress the protective effect of the clinically tested anti-SA monoclonal antibody in mice. Further evaluation of natural anti-Hla antibody competition with suvratoxumab is warranted.

Vaccination with immune-subdominant SA antigens circumvents interference

Our data above provided important insight into the complex interaction between SA-induced and vaccine-derived immunity and led us to hypothesize that vaccinating against immunologically subdominant antigens that do not naturally induce robust antibody titers may be a way to circumvent interference from immune imprinting. In support, we have previously shown that IsdB vaccination of mice pre-exposed to IsdB-deficient SA, and thus lacking IsdB-specific immunity, was protective compared to vaccination of mice pre-exposed to wild-type SA.[13]

To test our hypothesis, we evaluated vaccines that target subdominant antigens, which we define as SA proteins that naturally induced antibody titers in the lowest quartile even after 3× exposure to wild-type SA in mice (Figures 1E, 4A, and 4E). Among the ten antigens evaluated, murine SA infections consistently induced levels of anti-ClfA antibodies in the lowest quartile (Q1: 0.322 μg/mL) (Figure 5E). Vaccination of naive or SA-infected mice with alum-adjuvanted ClfA elicited high titers of specific antibodies that SA infection alone did not induce (Figure S6A) and resulted in anti-SA protection in both naive and SA-exposed mice (Figures 4B and S6B). *In vivo* adoptive serum transfer and *in vitro* OPK assays corroborated the protective role of the specific antibodies (Figures 4C, 4D, S6C, and S6D). Consistent with these findings, vaccination against two additional antigens from the panel, SdrE and EsxAB, which also induced low levels of humoral imprint after SA exposure, produced equivalent protection in naive and SA-experienced mice *in vivo* and in OPK assays (Figures 4E–4G, S6E, and S6F). Collectively, these findings are consistent with our hypothesis that targeting immune subdominant antigens could

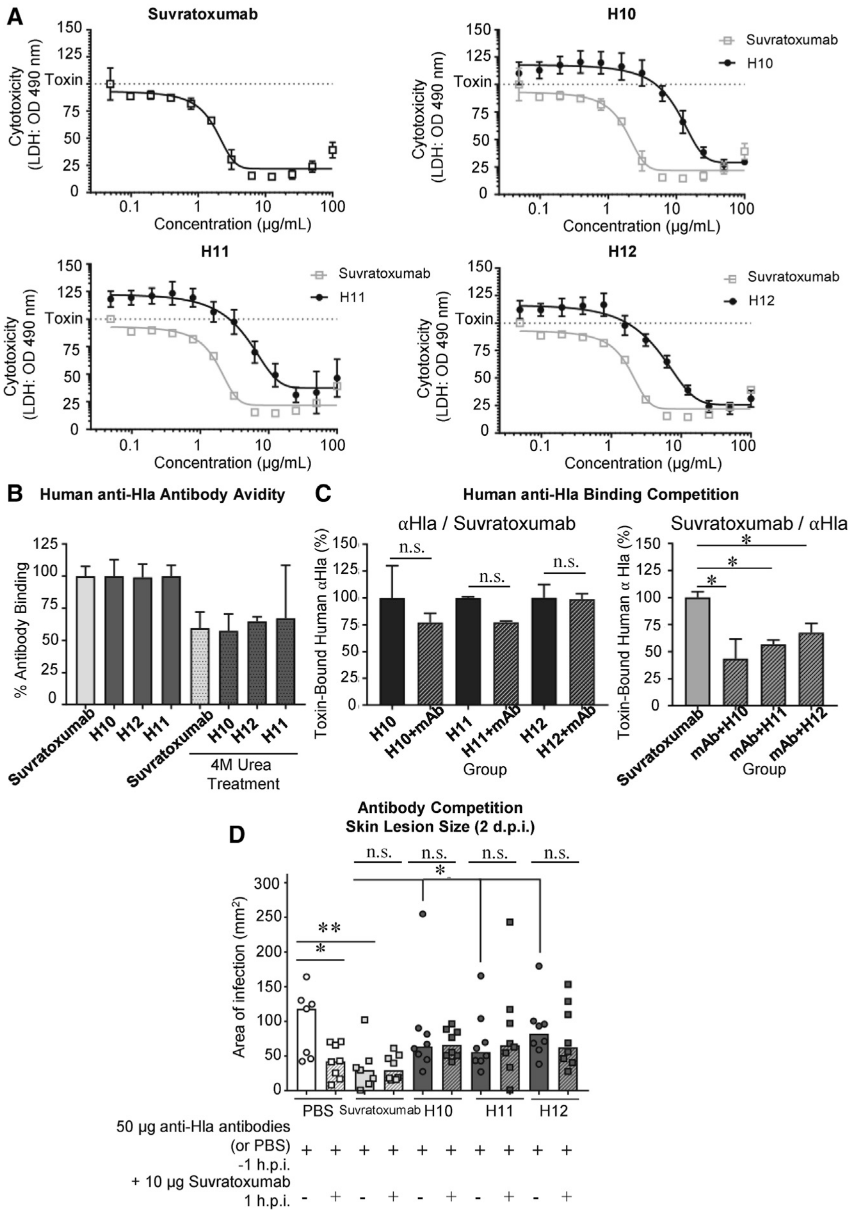

Figure 3. Pre-existing human anti-Hla antibodies blunt the efficacy of the anti-Hla monoclonal antibody suvratoxumab

(A) Neutralization of toxin-mediated THP-1 cytolysis using 0.05–100 µg purified anti-Hla human antibodies or suvratoxumab. Each point represents the mean of 4 technical replicates.

(B) Anti-Hla antibodies binding to recombinant Hla in the presence or absence of 4 M urea treatment. Bar corresponds to the mean of 6 technical replicates from two independent experiments.

(C) Left: retention of purified human anti-Hla antibody (10 µg/mL) binding to rHla after washing and addition of competing suvratoxumab (30 µg/mL) or PBS; right: retention of suvratoxumab (10 µg/mL) binding to rHla after washing and addition of competing purified human antibodies (30 µg/mL) or PBS. Bar corresponds to the mean of 3 technical replicates.

(D) Skin lesion size 2 dpi in CD-1 mice passively immunized with anti-Hla antibodies (50 µg/mouse), or PBS 1 h prior to infection, followed by suvratoxumab treatment (10 µg/mouse) or PBS 1 h post-infection. Each point represents an individual mouse. Bar corresponds to the median.

Error bars represent means ± SD; n.s., not significant, *$p < 0.05$, **$p < 0.01$, and ***$p < 0.001$; one-way ANOVA (B–D).

be a practical approach to circumventing interference in SA-exposed hosts.

Efficacy of anti-ClfA passive immunization is variably impacted by titer of pre-existing imprints

Based on the finding that low-level non-protective imprint does not interfere with active SA vaccination, we next asked how naturally variable levels of humoral imprint in human sera could impact the efficacy of monoclonal antibody therapies. To test this hypothesis, we evaluated competition between the human anti-ClfA monoclonal antibody tefibazumab and human sera with low to moderate levels of anti-ClfA antibodies, which reflects anti-ClfA antibody titers in healthy subjects and subjects who had invasive disease.[6,11] Tefibazumab was evaluated as an adjunctive therapeutic in a phase 2, randomized, double-blind, multicenter study for the treatment of patients with SA bacteremia. The trial with 60 enrolled patients failed to demonstrate a significant difference in composite clinical endpoint.[32]

For our study, we utilized whole human sera selected to reflect the varying levels of pre-existing anti-ClfA antibodies in humans (Figure 5A). We first evaluated antigen-binding competition between purified human antibodies and tefibazumab. We showed that at a ratio of 10:1, some human serum-derived anti-ClfA antibodies reduced tefibazumab binding to ClfA-coated plates (Figure 5B). Interestingly, serum sample H17, which has one of the lower titers of anti-ClfA antibodies in the group, was the most potent inhibitor of tefibazumab binding when compared at equimolar concentration to other serum anti-ClfA antibodies. To simulate tefibazumab treatment in hosts with pre-existing human anti-ClfA antibodies, we injected naive mice with whole human sera with the varying titers of anti-ClfA antibodies or the same sera that have been largely depleted of ClfA-specific antibodies (Figure 5A). We treated the mice with tefibazumab (300 µg) after 17 h and then infected the mice with SA after another hour. As shown in Figure 5C, tefibazumab was not protective when added to any of the human samples, suggesting the specific potency of human anti-ClfA antibody interference even in smaller quantities. After anti-ClfA antibody depletion, tefibazumab was protective in all samples except for sample H14 when compared to their corresponding untreated ClfA-depleted human sera (Figure 5D).

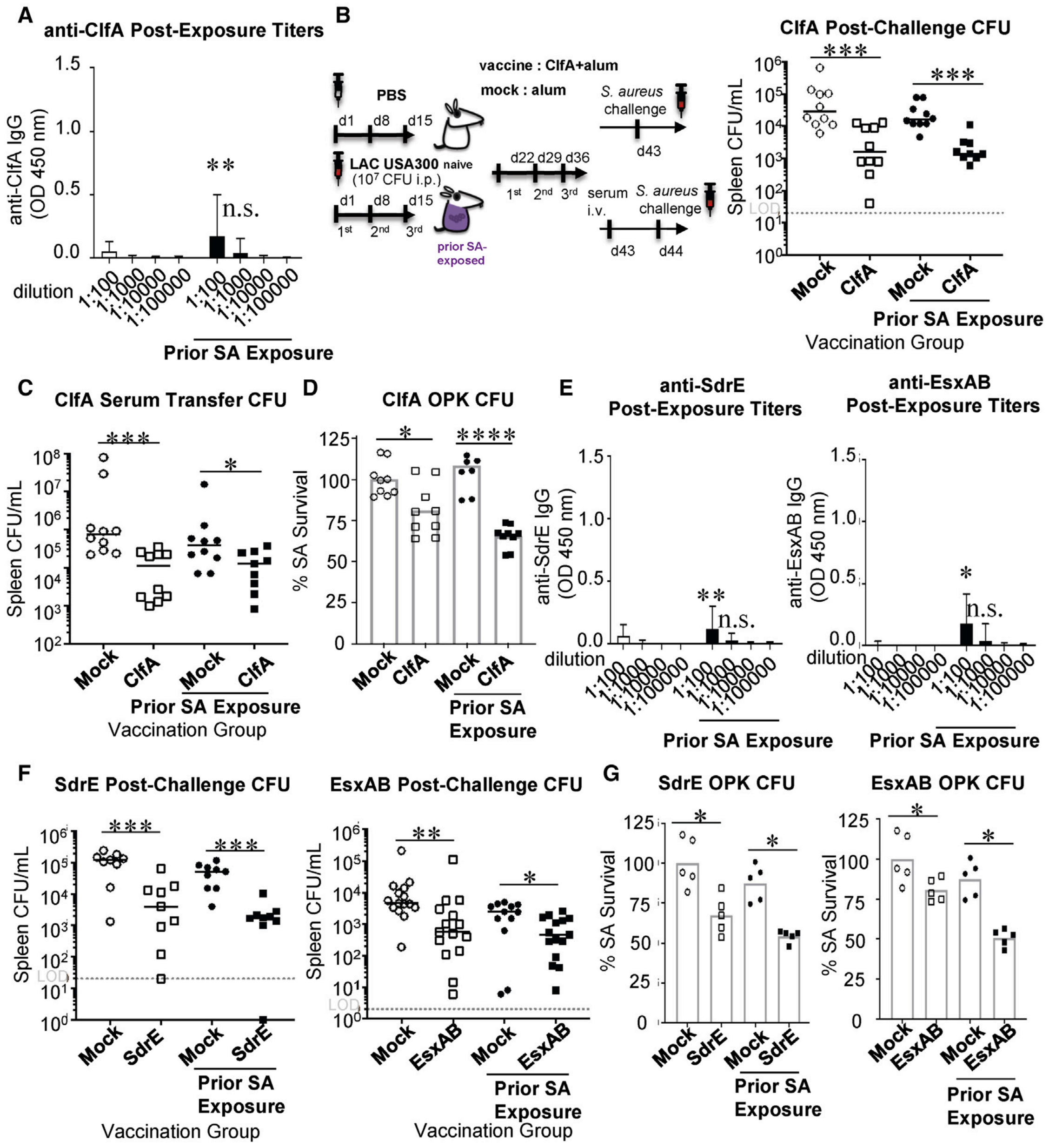

Figure 4. Subdominant SA CWAs induce protective immunity in SA-experienced mice

(A and E) ClfA-, SdrE-, and EsxAB-specific titers from n = 10 naive and SA-experienced mice 7 days after the last SA exposure. Bar corresponds to the median. Error bar corresponds to the range.

(B and F) Schematic of experimental design: naive mice were injected i.p. with PBS or 2.5 × 10^7 CFU SA once every 7 days × 3 weeks. Seven days after the last injection, SA-infected (or PBS-injected) mice were immunized with Alum or Alum/ClfA i.p. weekly × 3. Seven days after the last immunization, the mice challenged with SA or sera were harvested and injected i.v. into naive recipient mice, followed by challenge with 2.5 × 10^7 CFU SA. Bacterial burdens in spleen and kidneys were enumerated after 24 h. Post-challenge bacterial burden in spleen of mock- or ClfA-, SdrE-, or EsxAB-vaccinated naive or SA-experienced mice is shown. Bar corresponds to the median.

(C) Post-challenge bacterial burden in spleen of mice adoptively transferred serum from mock- or ClfA-vaccinated naive or SA-experienced mice.

(D and G) *In vitro* assessment of relative protective serum anti-ClfA, anti-SdrE, and anti-EsxAB antibody function by OPK. Results are normalized to mock (mIgG) control. C57BL/6 mice were used.

Bar represents group median; error bars represent means ± SD (A–E). Each point represents an individual mouse (B, C, and F); bar corresponds to the median, and dashed lines indicate the limit of detection (LOD) (B, C, and F). Bar represents the mean (D and G). n.s., not significant, $^*p < 0.05$, $^{**}p < 0.01$, and $^{***}p < 0.001$; Student's t test (A and E) or one-way ANOVA (B–D, F, and G).

CellPress
OPEN ACCESS

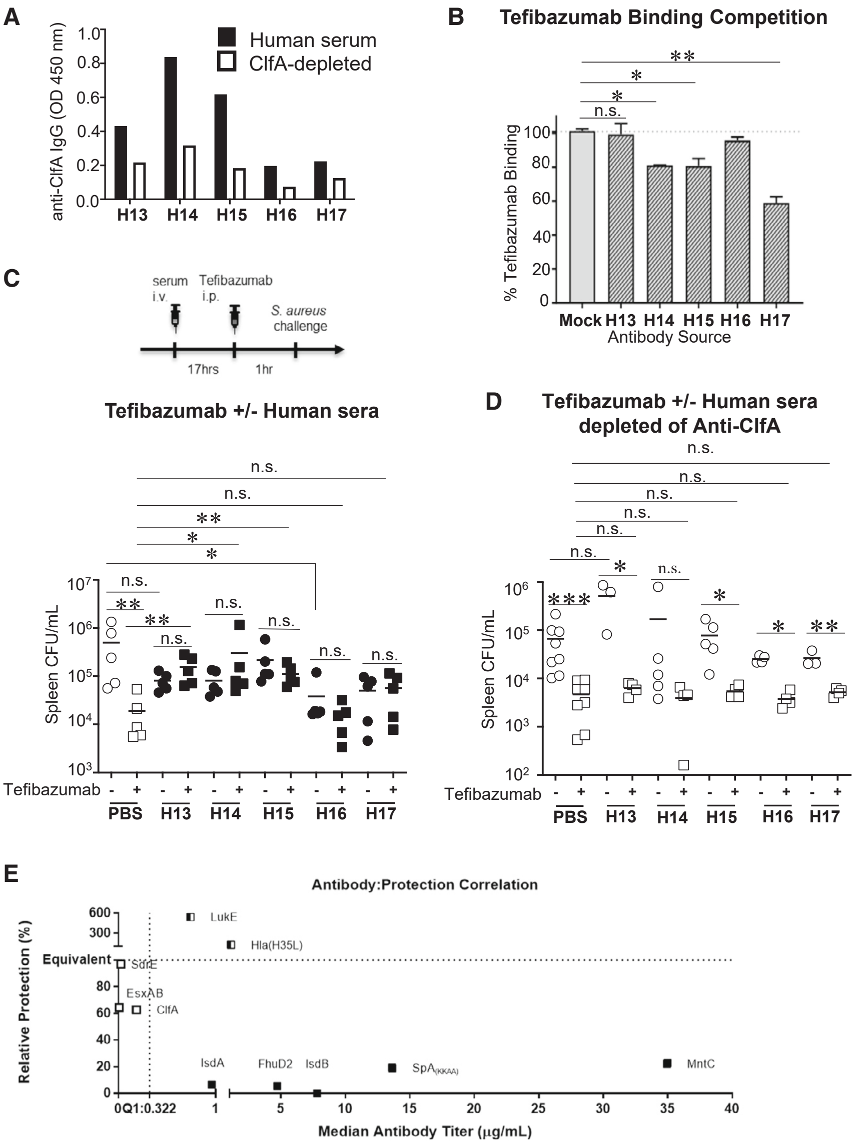

Figure 5. Naturally occurring human anti-ClfA antibody levels variably impact the anti-ClfA monoclonal antibody tefibazumab

(A) Anti-ClfA IgG titer before and after depletion through a ClfA-antibody-adsorbing column.

(B) Tefibazumab (1 μm/mL) binding to recombinant ClfA in the presence or absence of competing purified anti-ClfA human antibodies (10 μg/mL). Bar corresponds to the mean of 3 technical replicates.

(C and D) Schematic of experiments. Naive mice were injected with 100 μL human sera or human sera depleted of ClfA antibodies i.v., administered 300 μg tefibazumab i.p. after 17 h, and then infected with SA 1 h after tefibazumab injection. Experiment using whole human sera (C) or human sera depleted of ClfA antibodies (D).

(E) Composite analysis of association between median antibody titer and vaccine-mediated protection in SA-experienced mice. The x axis represents the median antibody titer (μg/mL) 7 days after the last infection. The y axis represents relative protection (%) in vaccinated SA-experienced mice calculated as the ratio between vaccine efficacy in SA-experienced mice and vaccine efficacy in naive mice. C57BL/6 mice were used.

Bar represents group median; error bars represent means ± SD (B). Each point represents an individual mouse (C and D); bar corresponds to the median (C and D). n.s., not significant, *$p < 0.05$, **$p < 0.01$, and ***$p < 0.001$; Student's t test (C and D) or one-way ANOVA (B, C, and D).

Notably, in direct comparison to mice given tefibazumab alone (in PBS) versus tefibazumab plus whole human sera, we showed that tefibazumab lost protection when added to moderate-to-high-titer serum samples (H13–H15) but not when the monoclonal antibody was added to low-titer serum samples (H16–H17) (Figure 5C). With anti-ClfA depletion, tefibazumab protection was unchanged with or without serum samples (Figure 5D). Altogether, the data suggest that human anti-ClfA antibodies can suppress tefibazumab protective efficacy.

DISCUSSION

In this study, we set out to broadly characterize the humoral imprints induced by prior SA infection with the intent of identifying strategies that avoid their adverse effect on immunization. We measured antigen-specific IgG titers and their corresponding protective efficacy by OPK assay for CWAs and by cytotoxicity assay for toxins. Remarkably, we showed that the titer and protective function of the humoral imprint alone predicted the efficacy of antigen-specific vaccines tested. These data are consistent with the published outcome of the SA clinical trials, for which the naive mouse model have provided little to no predictive value.

We showed that active vaccination against CWAs, which induce non-protective imprints, are ineffective in SA pre-infected mice and that active vaccination against toxins, which induce protective imprints, are effective. We defined the titer of antibody imprint that induced interference based on an arbitrary threshold above the first quartile (Q1: 0.32 μg/mL). Above that level, we did not see a direct correlation between titers and levels of non-protection (Figure 5E). Hence, we favor the interpretation that interference is bimodal and that a level of non-protective antibody above a threshold is more likely to predict vaccine failure.

The mechanism of vaccine interference induced by SA bears similarity to the original antigenic sin hypothesis[12] that was proposed to explain poor vaccine efficacy to influenza strains that have undergone seasonal drift. Failure of influenza vaccine is proposed to result from preferential recall of imprints that fail to strongly cross-react with newer influenza strains. In the case of SA, there is no antigenic drift. Failure of vaccines comes from the recall of non-protective imprints against the same

antigen.[13] We showed that these non-protective antibodies could blunt efficacy of protective antibodies by competition. Hence, pre-existing antibodies have the potential to dampen or block the effect therapeutic anti-SA monoclonal antibodies. In our published IsdB study, we have shown that the effect of antibody interference could be dramatic *in vivo* (when applying protective murine and non-protective human anti-IsdB antibodies at a ratio of 10 protective to 1 non-protective antibody), despite non-impressive *vitro* competitive binding data (40% displacement of protective antibodies using a reverse ratio of 1 protective to 10 non-protective antibodies).[13] Here, we provided additional evidence of human serum interference with two monoclonal antibodies that failed clinical trials, suvratoxumab and tefibazumab monoclonal antibodies.[30,32] Notably, human anti-ClfA displacement of tefibazumab, like IsdB antibodies, is unimpressive *in vitro*, but inhibition of tefibazumab effect was observed using a ratio of 300 μg tefibazumab to $\sim$2 μg anti-ClfA antibodies (based on estimation of serum anti-ClfA concentrations from Figure 1A human serum anti-ClfA median concentration). Although the *in vivo* experimental condition could in principle be construed to mimic human trial ratio, caution must be exercised in the interpretation of clinical relevance of the data. It also remains unclear why there is a lack of correlation between *in vitro* and *in vivo* competition.

Another hypothesis that was tested in our study was that successful vaccines could be developed in SA-immune subjects by targeting antigens that show no or modest prior humoral imprints. Targeting of subdominant antigenic domains has been proposed as a therapeutic strategy in infectious disease models[41]; however, due to the unclear immunogenicity of these antigens in humans, clinical vaccine approaches have, for the most part, targeted immunodominant antigens that already demonstrate robust immunogenicity. Immunodominant SA CWA vaccines appear to be different in that they induce non-protective antibodies in SA-immune individuals, which result in vaccine failure. Hence, different strategies are needed for SA vaccine development. Our study showed that targeting subdominant antigens and toxins are both likely effective active vaccine strategies. Teymournejad et al. have recently shown in a skin infection model that anti-staphylococcal toxin vaccines induce primarily a T cell-mediated response that, although protective, is suboptimal because of pre-existing, less-protective imprint.[42] Aside from the differences in prior infection model (i.p. versus subcutaneous) and the branch of adaptive immune response elicited, the two studies are largely compatible in that, irrespective of the effect of the prior imprints, anti-toxin active vaccines were protective in SA-experienced mice. In contrast to the protective active anti-toxin vaccine strategy, we showed that the presence of pre-existing anti-Hla antibodies could lead to failure of anti-Hla monoclonal antibody therapy. Although both subdominant CWAs and toxins could induce vaccine protection, neutralization of toxins could prove to be more effective during acute infection, whereas targeting of CWAs could, theoretically, be more important after SA adaptation to hosts in chronic diseases. Hence, there could be utility in targeting both types of antigens for the different types of infection. Another point of consideration is the level of pre-existing anti-toxin antibody titers in human adults, which is quite high and which we presume would confer a finite ceiling of protection. Hence, we speculate that vaccination against toxins would be beneficial in infants and children but significantly less so in older adults. Based on this consideration, subdominant antigens may prove to be more attractive vaccine targets in elderly subjects.

Although ClfA, SdrE, and EsxAB were annotated as subdominant antigens in our study, two of these antigens, ClfA and SdrE, are actually immunodominant glycoproteins.[43,44] It could be speculated that the glycan components that generate abundant antibody responses are non-protective and serve as decoys to minimize host immune response to the subdominant protein component of the antigen, which would be an important question to study. This study used proteins that were not glycosylated, and therefore only IgGs reactive with the protein backbone were isolated and used. Accordingly, we define dominance or subdominance in the study based on the bare protein backbone of the SA antigen. Within that narrower scope, immune imprints appear to predict the outcome of SA vaccination.

Among the CWAs tested in this study is the SA virulence factor SpA, a potent immunomodulatory protein with significant impact on B cell development and antibody functions through binding to Fcγ and VH3.[45–51] Binding to Fcγ interferes with opsonophagocytic functions of specific antibodies, whereas binding to VH3 leads to B cell superantigenic activity. The use of SpA_{KKAA}, which has minimal cross-reactivity to Fcγ and VH3, abrogates these SpA-related activities and leads to protection in mice. Our *in vivo* investigations of SpA as a vaccine candidate in SA-immune host could be confounded by these intrinsic functions as a B cell-antagonizing virulence determinant,[52,53] and SpA_{KKAA} vaccine was barely non-efficacious in SA-exposed mice. The *in vitro* data suggest that SpA could be viewed as an immunodominant CWA that follows the imprinting hypothesis. Immune imprinting as it relates to SpA deserves further studies.

Findings from vaccination studies conducted on SA-experienced mice must be scrutinized in a human-specific context. As shown, immunogenicity of SA antigens can significantly differ between humans and mice; MntC-specific antibody levels, for example, are robust and persistent after a single SA infection in C57BL/6 mice but are modest in human sera. In addition, our study examined only ten SA vaccine antigens and nine human subjects, and conclusions from our findings clearly need to be further validated. Ultimate corroboration of the presented concepts will require analysis and validation from successful SA trials, which do not yet exist.

The universal failure in staphylococcal vaccinology has been a long-standing conundrum that we propose is rooted in flawed modeling and design. Recent investigations on the effects of prior microbial exposures on efficacy of therapeutics highlight the interaction between pre-existing immunity and subsequent immune induction.[54,55] These findings bolster the argument that the integration of clinical human data into basic animal models is needed if we are to successfully model the highly complex immune environment surrounding vaccinations. In our present study, we have presented an alternate approach on how we could re-evaluate failed vaccines and develop successful strategies based on the new framework.

Limitations of the study

There are several limitations to our study. Prior exposure to SA consisted of three i.p. mouse infections, which do not mimic human exposure. The experiments that demonstrate inhibition of tefibazumab and suvratoxumab do not closely follow the clinical trial designs. The vaccine doses used in mice do not follow those used in human trials. Our study demonstrates general principles of imprinting effect on vaccine and needs to be interpreted with caution when extrapolated to human data.

STAR★METHODS

Detailed methods are provided in the online version of this paper and include the following:

- KEY RESOURCES TABLE
- RESOURCE AVAILABILITY
  - Lead contact
  - Materials availability
  - Data and code availability
- EXPERIMENTAL MODEL AND SUBJECT DETAILS
  - Ethics statement
  - Murine models of *S. aureus* prior exposure and infection
  - Bacteria
- METHOD DETAILS
  - Cloning and protein expression
  - Reverse: LukE(5′-CAGAATTCGCAAGCTTTAATTATGTCCTTTCACTTT-PRIMER-3′)
  - Antibody measurements (ELISA)
  - Antibody purification
  - Antibody competition
  - Immunization with vaccine proteins
  - Adoptive transfer of serum or IgG
  - Opsonophagocytic killing assay/SA survival assay
  - Culture of THP-1 cells
  - Cytotoxicity/toxin neutralization assay
  - Alpha toxin antibody avidity assay
  - Adoptive transfer of sera or purified antibodies
  - Flow cytometric analysis of antibody binding to *S. aureus*
  - SDS-PAGE and immunoblot
- QUANTIFICATION AND STATISTICAL ANALYSIS

SUPPLEMENTAL INFORMATION

Supplemental information can be found online at https://doi.org/10.1016/j.xcrm.2023.101360.

ACKNOWLEDGMENTS

We thank Dr. L.J. Tovar-Padua and Dr. E.C. Ulloa for aiding in the collection of human serum from healthy volunteers. The study was funded by National Institutes of Health grants R01AI127406 (G.Y.L.), R01AI144694 (G.Y.L.), and R01AI181321 (G.Y.L.).

AUTHOR CONTRIBUTIONS

J.R.C. and C.-M.T. were responsible for designing and conducting most experiments, with expert guidance from G.Y.L. D.T. and B.L. helped with recombinant protein cloning, expression, and purification. D.T., C.G., X.D., and I.A.H. participated in the *in vivo* vaccination and infection experiments. J.R.C. and G.Y.L. wrote the manuscript, with all authors providing significant input.

DECLARATION OF INTERESTS

G.Y., C.-M.T., and I.A.H. have received a research grant from Pfizer to study SA vaccines.

Received: January 15, 2023
Revised: August 15, 2023
Accepted: December 10, 2023
Published: January 16, 2024

REFERENCES

1. CDC (2018). Biggest Threats and Data. https://www.cdc.gov/drugresistance/biggest_threats.html?CDC_AA_refVal=https%3A%2F%2Fwww.cdc.gov%2Fdrugresistance%2Fthreat-report-2013%2Findex.html.
2. Fowler, V.G., and Proctor, R.A. (2014). Where does a Staphylococcus aureus vaccine stand? Clin. Microbiol. Infect. *20*, 66–75.
3. Miller, L.S., Fowler, V.G., Shukla, S.K., Rose, W.E., and Proctor, R.A. (2020). Development of a vaccine against Staphylococcus aureus invasive infections: Evidence based on human immunity, genetics and bacterial evasion mechanisms. FEMS Microbiol. Rev. *44*, 123–153.
4. Meyer, T.C., Michalik, S., Holtfreter, S., Weiss, S., Friedrich, N., Völzke, H., Kocher, T., Kohler, C., Schmidt, F., Bröker, B.M., and Völker, U. (2021). A Comprehensive View on the Human Antibody Repertoire Against Staphylococcus aureus Antigens in the General Population. Front. Immunol. *12*, 651619.
5. Radke, E.E., Brown, S.M., Pelzek, A.J., Fulmer, Y., Hernandez, D.N., Torres, V.J., Thomsen, I.P., Chiang, W.K., Miller, A.O., Shopsin, B., and Silverman, G.J. (2018). Hierarchy of human IgG recognition within the Staphylococcus aureus immunome. Sci. Rep. *8*, 13296.
6. Colque-Navarro, P., Jacobsson, G., Andersson, R., Flock, J.I., and Möllby, R. (2010). Levels of antibody against 11 Staphylococcus aureus antigens in a healthy population. Clin. Vaccine Immunol. *17*, 1117–1123.
7. Zorman, J.K., Esser, M., Raedler, M., Kreiswirth, B.N., Ala'Aldeen, D.A.A., Kartsonis, N., Smugar, S.S., Anderson, A.S., McNeely, T., and Arduino, J.M. (2013). Naturally occurring IgG antibody levels to the Staphylococcus aureus protein IsdB in humans. Hum. Vaccin. Immunother. *9*, 1857–1864.
8. Wu, Y., Liu, X., Akhgar, A., Li, J.J., Mok, H., Sellman, B.R., Yu, L., Roskos, L.K., Esser, M.T., and Ruzin, A. (2018). Prevalence of IgG and Neutralizing Antibodies against Staphylococcus aureus Alpha-Toxin in Healthy Human Subjects and Diverse Patient Populations. Infect. Immun. *86*, e00671–17.
9. Schulz, D., Grumann, D., Trübe, P., Pritchett-Corning, K., Johnson, S., Reppschläger, K., Gumz, J., Sundaramoorthy, N., Michalik, S., Berg, S., et al. (2017). Laboratory Mice Are Frequently Colonized with Staphylococcus aureus and Mount a Systemic Immune Response-Note of Caution for In vivo Infection Experiments. Front. Cell. Infect. Microbiol. *7*, 152.
10. Tsai, C.M., Soper, N., Bennett, M., Fallon, J.K., Michell, A.R., Alter, G., Liu, G.Y., and Thomsen, I. (2021). Adoptive Transfer of Serum Samples From Children With Invasive Staphylococcal Infection and Protection Against Staphylococcus aureus Sepsis. J. Infect. Dis. *223*, 1222–1231.
11. Jacobsson, G., Colque-Navarro, P., Gustafsson, E., Andersson, R., and Möllby, R. (2010). Antibody responses in patients with invasive Staphylococcus aureus infections. Eur. J. Clin. Microbiol. Infect. Dis. *29*, 715–725.
12. Francis, T.J. (1960). On the Doctrine of Original Antigenic Sin. Proc. Am. Phil. Soc. *104*, 572–578.
13. Tsai, C.M., Caldera, J.R., Hajam, I.A., Chiang, A.W.T., Tsai, C.H., Li, H., Díez, M.L., Gonzalez, C., Trieu, D., Martins, G.A., et al. (2022). Non-protective immune imprint underlies failure of Staphylococcus aureus IsdB vaccine. Cell Host Microbe *30*, 1163–1172.e6.

14. Kim, H.K., DeDent, A., Cheng, A.G., McAdow, M., Bagnoli, F., Missiakas, D.M., and Schneewind, O. (2010). IsdA and IsdB antibodies protect mice against Staphylococcus aureus abscess formation and lethal challenge. Vaccine *28*, 6382–6392.

15. Stranger-Jones, Y.K., Bae, T., and Schneewind, O. (2006). Vaccine assembly from surface proteins of Staphylococcus aureus. Proc. Natl. Acad. Sci. USA *103*, 16942–16947.

16. Mishra, R.P.N., Mariotti, P., Fiaschi, L., Nosari, S., Maccari, S., Liberatori, S., Fontana, M.R., Pezzicoli, A., De Falco, M.G., Falugi, F., et al. (2012). Staphylococcus aureus FhuD2 is involved in the early phase of staphylococcal dissemination and generates protective immunity in mice. J. Infect. Dis. *206*, 1041–1049.

17. Bagnoli, F., Fontana, M.R., Soldaini, E., Mishra, R.P.N., Fiaschi, L., Cartocci, E., Nardi-Dei, V., Ruggiero, P., Nosari, S., De Falco, M.G., et al. (2015). Vaccine composition formulated with a novel TLR7-dependent adjuvant induces high and broad protection against Staphylococcus aureus. Proc. Natl. Acad. Sci. USA *112*, 3680–3685.

18. Begier, E., Seiden, D.J., Patton, M., Zito, E., Severs, J., Cooper, D., Eiden, J., Gruber, W.C., Jansen, K.U., Anderson, A.S., and Gurtman, A. (2017). SA4Ag, a 4-antigen Staphylococcus aureus vaccine, rapidly induces high levels of bacteria-killing antibodies. Vaccine *35*, 1132–1139.

19. Inoue, M., Yonemura, T., Baber, J., Shoji, Y., Aizawa, M., Cooper, D., Eiden, J., Gruber, W.C., Jansen, K.U., Anderson, A.S., and Gurtman, A. (2018). Safety, tolerability, and immunogenicity of a novel 4-antigen Staphylococcus aureus vaccine (SA4Ag) in healthy Japanese adults. Hum. Vaccin. Immunother. *14*, 2682–2691.

20. Li, X., Wang, X., Thompson, C.D., Park, S., Park, W.B., and Lee, J.C. (2016). Preclinical Efficacy of Clumping Factor A in Prevention of Staphylococcus aureus Infection. mBio *7*. e02232-e02215.

21. Scully, I.L., Timofeyeva, Y., Keeney, D., Matsuka, Y.V., Severina, E., McNeil, L.K., Nanra, J., Hu, G., Liberator, P.A., Jansen, K.U., and Anderson, A.S. (2015). Demonstration of the preclinical correlate of protection for Staphylococcus aureus clumping factor A in a murine model of infection. Vaccine *33*, 5452–5457.

22. Kim, H.K., Cheng, A.G., Kim, H.Y., Missiakas, D.M., and Schneewind, O. (2010). Nontoxigenic protein A vaccine for methicillin-resistant Staphylococcus aureus infections in mice. J. Exp. Med. *207*, 1863–1870.

23. Giersing, B.K., Dastgheyb, S.S., Modjarrad, K., and Moorthy, V. (2016). Status of vaccine research and development of vaccines for Staphylococcus aureus. Vaccine *34*, 2962–2966.

24. Shinefield, H., Black, S., Fattom, A., Horwith, G., Rasgon, S., Ordonez, J., Yeoh, H., Law, D., Robbins, J.B., Schneerson, R., et al. (2002). Use of a Staphylococcus aureus conjugate vaccine in patients receiving hemodialysis. N. Engl. J. Med. *346*, 491–496.

25. Fowler, V.G., Allen, K.B., Moreira, E.D., Moustafa, M., Isgro, F., Boucher, H.W., Corey, G.R., Carmeli, Y., Betts, R., Hartzel, J.S., et al. (2013). Effect of an investigational vaccine for preventing Staphylococcus aureus infections after cardiothoracic surgery: a randomized trial. JAMA *309*, 1368–1378.

26. gov, C.T. (2020). Safety and Efficacy of SA4Ag Vaccine in Adults Having Elective Open Posterior Spinal Fusion Procedures with Multilevel Instrumentation (STRIVE).

27. Tam, K., Lacey, K.A., Devlin, J.C., Coffre, M., Sommerfield, A., Chan, R., O'Malley, A., Koralov, S.B., Loke, P., and Torres, V.J. (2020). Targeting leukocidin-mediated immune evasion protects mice from Staphylococcus aureus bacteremia. J. Exp. Med. *217*, e20190541.

28. Adhikari, R.P., Thompson, C.D., Aman, M.J., and Lee, J.C. (2016). Protective efficacy of a novel alpha hemolysin subunit vaccine (AT62) against Staphylococcus aureus skin and soft tissue infections. Vaccine *34*, 6402–6407.

29. Zeng, H., Yang, F., Feng, Q., Zhang, J., Gu, J., Jing, H., Cai, C., Xu, L., Yang, X., Xia, X., et al. (2020). Rapid and broad immune efficacy of a recombinant five-antigen vaccine against staphylococcus aureus infection in animal models. Vaccines *8*.

30. François, B., Jafri, H.S., Chastre, J., Sánchez-García, M., Eggimann, P., Dequin, P.F., Huberlant, V., Viña Soria, L., Boulain, T., Bretonnière, C., et al. (2021). Efficacy and safety of suvratoxumab for prevention of Staphylococcus aureus ventilator-associated pneumonia (SAATELLITE): a multicentre, randomised, double-blind, placebo-controlled, parallel-group, phase 2 pilot trial. Lancet Infect. Dis. *21*, 1313–1323.

31. Yu, W., Yao, D., Yu, S., Wang, X., Li, X., Wang, M., Liu, S., Feng, Z., Chen, X., Li, W., et al. (2018). Protective humoral and CD4(+) T cellular immune responses of Staphylococcus aureus vaccine MntC in a murine peritonitis model. Sci. Rep. *8*, 3580.

32. Weems, J.J., Jr., Steinberg, J.P., Filler, S., Baddley, J.W., Corey, G.R., Sampathkumar, P., Winston, L., John, J.F., Kubin, C.J., Talwani, R., et al. (2006). Phase II, randomized, double-blind, multicenter study comparing the safety and pharmacokinetics of tefibazumab to placebo for treatment of Staphylococcus aureus bacteremia. Antimicrob. Agents Chemother. *50*, 2751–2755.

33. Magyarics, Z., Leslie, F., Bartko, J., Rouha, H., Luperchio, S., Schörgenhofer, C., Schwameis, M., Derhaschnig, U., Lagler, H., Stiebellehner, L., et al. (2019). Randomized, Double-Blind, Placebo-Controlled, Single-Ascending-Dose Study of the Penetration of a Monoclonal Antibody Combination (ASN100) Targeting Staphylococcus aureus Cytotoxins in the Lung Epithelial Lining Fluid of Healthy Volunteers. Antimicrob. Agents Chemother. *63*, e00350–19.

34. Chen, X., Shi, M., Tong, X., Kim, H.K., Wang, L.X., Schneewind, O., and Missiakas, D. (2020). Glycosylation-dependent opsonophagocytic activity of staphylococcal protein A antibodies. Proc. Natl. Acad. Sci. USA *117*, 22992–23000.

35. Adhikari, R.P., Kort, T., Shulenin, S., Kanipakala, T., Ganjbaksh, N., Roghmann, M.C., Holtsberg, F.W., and Aman, M.J. (2015). Antibodies to S. aureus LukS-PV Attenuated Subunit Vaccine Neutralize a Broad Spectrum of Canonical and Non-Canonical Bicomponent Leukotoxin Pairs. PLoS One *10*, e0137874.

36. Fritz, S.A., Tiemann, K.M., Hogan, P.G., Epplin, E.K., Rodriguez, M., Al-Zubeidi, D.N., Bubeck Wardenburg, J., and Hunstad, D.A. (2013). A serologic correlate of protective immunity against community-onset Staphylococcus aureus infection. Clin. Infect. Dis. *56*, 1554–1561.

37. Sharma-Kuinkel, B.K., Tkaczyk, C., Bonnell, J., Yu, L., Tovchigrechko, A., Tabor, D.E., Park, L.P., Ruffin, F., Esser, M.T., Sellman, B.R., et al. (2019). Associations of pathogen-specific and host-specific characteristics with disease outcome in patients with Staphylococcus aureus bacteremic pneumonia. Clin. Transl. Immunology *8*, e01070.

38. Bubeck Wardenburg, J., and Schneewind, O. (2008). Vaccine protection against Staphylococcus aureus pneumonia. J. Exp. Med. *205*, 287–294.

39. Adhikari, R.P., Ajao, A.O., Aman, M.J., Karauzum, H., Sarwar, J., Lydecker, A.D., Johnson, J.K., Nguyen, C., Chen, W.H., and Roghmann, M.C. (2012). Lower antibody levels to Staphylococcus aureus exotoxins are associated with sepsis in hospitalized adults with invasive S. aureus infections. J. Infect. Dis. *206*, 915–923.

40. Christensson, B., Hedström, S.A., and Kronvall, G. (1983). Antibody response to alpha- and betahemolysin from Staphylococcus aureus in patients with staphylococcal infections and in normals. Acta Pathol. Microbiol. Immunol. Scand. B *91*, 351–356.

41. Lindahl, G. (2020). Subdominance in Antibody Responses: Implications for Vaccine Development. Microbiol. Mol. Biol. Rev. *85*, e00078–20.

42. Teymournejad, O., Li, Z., Beesetty, P., Yang, C., and Montgomery, C.P. (2023). Toxin expression during Staphylococcus aureus infection imprints host immunity to inhibit vaccine efficacy. NPJ Vaccines *8*, 3.

43. Hazenbos, W.L.W., Kajihara, K.K., Vandlen, R., Morisaki, J.H., Lehar, S.M., Kwakkenbos, M.J., Beaumont, T., Bakker, A.Q., Phung, Q., Swem, L.R., et al. (2013). Novel staphylococcal glycosyltransferases SdgA and SdgB mediate immunogenicity and protection of virulence-associated cell wall proteins. PLoS Pathog. *9*, e1003653.

44. Thomer, L., Becker, S., Emolo, C., Quach, A., Kim, H.K., Rauch, S., Anderson, M., Leblanc, J.F., Schneewind, O., Faull, K.F., and Missiakas, D. (2014). N-acetylglucosaminylation of serine-aspartate repeat proteins promotes Staphylococcus aureus bloodstream infection. J. Biol. Chem. *289*, 3478–3486.

45. Forsgren, A., and Quie, P.G. (1974). Effects of staphylococcal protein A on heat labile opsonins. J. Immunol. *112*, 1177–1180.

46. Forsgren, A., and Sjöquist, J. (1966). "Protein A" from S. aureus. I. Pseudo-immune reaction with human gamma-globulin. J. Immunol. *97*, 822–827.

47. Goodyear, C.S., and Silverman, G.J. (2003). Death by a B cell superantigen: In vivo VH-targeted apoptotic supraclonal B cell deletion by a Staphylococcal Toxin. J. Exp. Med. *197*, 1125–1139.

48. Keener, A.B., Thurlow, L.T., Kang, S., Spidale, N.A., Clarke, S.H., Cunnion, K.M., Tisch, R., Richardson, A.R., and Vilen, B.J. (2017). Staphylococcus aureus Protein A Disrupts Immunity Mediated by Long-Lived Plasma Cells. J. Immunol. *198*, 1263–1273.

49. Kim, H.K., Falugi, F., Thomer, L., Missiakas, D.M., and Schneewind, O. (2015). Protein A suppresses immune responses during Staphylococcus aureus bloodstream infection in guinea pigs. mBio *6*, e02369–14.

50. Kobayashi, S.D., and DeLeo, F.R. (2013). Staphylococcus aureus protein A promotes immune suppression. mBio *4*. e00764-e00713.

51. Silverman, G.J., Cary, S.P., Dwyer, D.C., Luo, L., Wagenknecht, R., and Curtiss, V.E. (2000). A B cell superantigen-induced persistent "Hole" in the B-1 repertoire. J. Exp. Med. *192*, 87–98.

52. Tsai, C.M., Caldera, J., Hajam, I.A., and Liu, G.Y. (2023). Toward an effective Staphylococcus vaccine: why have candidates failed and what is the next step? Expert Rev. Vaccines *22*, 207–209.

53. Tsai, C.M., Hajam, I.A., Caldera, J.R., and Liu, G.Y. (2022). Integrating complex host-pathogen immune environments into S. aureus vaccine studies. Cell Chem. Biol. *29*, 730–740.

54. Rosshart, S.P., Herz, J., Vassallo, B.G., Hunter, A., Wall, M.K., Badger, J.H., McCulloch, J.A., Anastasakis, D.G., Sarshad, A.A., Leonardi, I., et al. (2019). Laboratory mice born to wild mice have natural microbiota and model human immune responses. Science *365*, eaaw4361.

55. Fiege, J.K., Block, K.E., Pierson, M.J., Nanda, H., Shepherd, F.K., Mickelson, C.K., Stolley, J.M., Matchett, W.E., Wijeyesinghe, S., Meyerholz, D.K., et al. (2021). Mice with diverse microbial exposure histories as a model for preclinical vaccine testing. Cell Host Microbe *29*, 1815–1827.e6.

56. Paterson, M.J., Caldera, J.R., Nguyen, C., Sharma, P., Castro, A.M., Kolar, S.L., Tsai, C.M., Limon, J.J., Becker, C.A., Martins, G.A., et al. (2020). Harnessing antifungal immunity in pursuit of a Staphylococcus aureus vaccine strategy. PLoS Pathog. *16*, e1008733.

57. Thomer, L., Emolo, C., Thammavongsa, V., Kim, H.K., McAdow, M.E., Yu, W., Kieffer, M., Schneewind, O., and Missiakas, D. (2016). Antibodies against a secreted product of Staphylococcus aureus trigger phagocytic killing. J. Exp. Med. *213*, 293–301.

58. Dekkers, G., Bentlage, A.E.H., Stegmann, T.C., Howie, H.L., Lissenberg-Thunnissen, S., Zimring, J., Rispens, T., and Vidarsson, G. (2017). Affinity of human IgG subclasses to mouse Fc gamma receptors. mAbs *9*, 767–773.

59. Craven, R.R., Gao, X., Allen, I.C., Gris, D., Bubeck Wardenburg, J., McElvania-Tekippe, E., Ting, J.P., and Duncan, J.A. (2009). Staphylococcus aureus alpha-hemolysin activates the NLRP3-inflammasome in human and mouse monocytic cells. PLoS One *4*, e7446.

60. Hall, A.E., Domanski, P.J., Patel, P.R., Vernachio, J.H., Syribeys, P.J., Gorovits, E.L., Johnson, M.A., Ross, J.M., Hutchins, J.T., and Patti, J.M. (2003). Characterization of a protective monoclonal antibody recognizing Staphylococcus aureus MSCRAMM protein clumping factor A. Infect. Immun. *71*, 6864–6870.

STAR★METHODS

KEY RESOURCES TABLE

REAGENT or RESOURCE	SOURCE	IDENTIFIER
Experimental models: Organisms/strains		
Mus musculus C57BL/6	Charles river	Strain #C57BL/6NCrl
Mus musculus CD1	Charles river	Strain # CD-1® IGS
Primary cells		
Mouse neutrophils	Bone marrow from *Mus musculus* C57BL/6	N/A
Cell line		
THP-1	ATCC	TIB-202™
Bacterial strains		
Staphylococcus aureus: LAC (USA300)	Dr. Binh Diep	N/A
Staphylococcus aureus: JE2	Dr. Victor Nizet	N/A
Staphylococcus aureus: ΔSpA (JE2)	Dr. Victor Nizet	N/A
E. coli BL21 (DE3)	NEB	Cat #C2527I
ClfA (BL21)	Dr. David Underhill	Cedars-Sinai
IsdA (BL21)	Dr. David Underhill	Cedars-Sinai
MntC (BL21)	Dr. David Underhill	Cedars-Sinai
FhuD2 (BL21)	Dr. David Underhill	Cedars-Sinai
Oligonucleotides		
Forward LukE: 5′-TAAGGCCTCTGTCGAAA ATACTAATATTGAAAATATTGGT-3′	Integrated DNA Technologies	N/A
Reverse LukE: 5′-CAGAATTCGCAAGCTTT AATTATGTCCTTTCACTTT-3′	Integrated DNA Technologies	N/A
Recombinant DNA		
pET-15b	Novagen	Cat # 69864
pET-6xHN	Takara	Cat # 631433
EsxAB	GenScript	N/A
SpA_{KKAA}	GenScript	N/A
alpha toxoid (Hla(H35L))	GenScript	N/A
Antibodies		
horseradish peroxidase (HRP)-conjugated goat anti-mouse IgG	Biolegend	Cat # 405306
horseradish peroxidase (HRP)-conjugated donkey anti-human IgG	Biolegend	Cat # 410902
Tefibazumab	Creative Biolabs	Cat # TAB-029
Suvratoxumab	Creative Biolabs	Cat # TAB-463CQ
FITC-conjugated anti-mouse IgG	Invitrogen	Cat # 31569
Chemicals, reagents and recombinant protein		
His60 Ni Superflow Resin	Takara	Cat # 635660
Aluminum hydroxide gel	InvivoGen	Cat # vac-alu-250
isopropyl-β-D-thiogalactoside (IPTG)	G-BIOSCIENCES	Cat # RC-063
Protein A from Staphylococcus aureus	Sigma-Aldrich	Cat #P6031
PierceTM Protein G Agarose	ThermoFisher Scientific	Cat # 20397
Amicon Ultra-15 Centrifugal Filter Unit	Millipore Sigma	Cat # UFC905024
α-Hemolysin from Staphylococcus aureus	Sigma	Cat #H9395-1MG
Recombinant LukE	Mayflower Bioscience	Cat # 0530-004

(*Continued on next page*)

Continued		
REAGENT or RESOURCE	SOURCE	IDENTIFIER
Recombinant LukD	Mayflower Bioscience	Cat # 783304
EZ-Link^{TM} Sulfo-NHS-Biotin	ThermoFisher Scientific	Cat # 21217
NHS-activated agarose	ThermoFisher Scientific	Cat # 2697
Baby Rabbit Complement	BIO-RAD	Cat #C12CA
Critical commercial assays		
MojoSort^{TM} Mouse neutrophils Isolation Kit	Biolegend	Cat # 480058
LDH cytotoxicity detection kit	Takara	Cat # MK401
Software and algorithms		
GraphPad Prism 10	GraphPad Software	https://www.graphpad.com/scientific-software/prism/
Microsoft Excel	Microsoft	https://www.microsoft.com/en-us/microsoft-365/excel
FlowJo (v.10.6.1)	FlowJo	https://www.flowjo.com/
bioRENDER	Biorender	https://www.biorender.com/

RESOURCE AVAILABILITY

Lead contact

Further information and requests for resources and reagents should be directed to and will be fulfilled by the lead contact, George Liu (gyliu@ucsd.edu).

Materials availability

All unique reagents generated in this study are available from the lead contact without restriction.

Data and code availability

All data reported in this paper will be shared by the lead contact upon request. This paper does not report the original code. Any additional information required to reanalyze the data reported in this paper is available from the lead contact upon request.

EXPERIMENTAL MODEL AND SUBJECT DETAILS

Ethics statement

Mouse studies were reviewed and approved by the Institutional Animal Care and Use Committee. Mouse experiments were conducted in accordance with regulations and recommendations on animal experiments cited by the Animal Care Programs at University of California, San Diego, and Cedars-Sinai Medical Center. Experimentations using human blood were approved by the UCSD Human Research Protection Program. Prior informed consents were obtained from the human subjects. Experimental protocols were approved by the UCSD Biosafety Committee.

Murine models of *S. aureus* prior exposure and infection

6–8-week-old female C57BL/6 or CD1 mice were purchased from Charles Rivers Laboratories (Wilmington, MA). For intraperitoneal (i.p.) injections, procedures were performed under manual restraint. For i.v. and intradermal (i.d.) injections, procedures were performed under isoflurane anesthesia with continuous oxygenation. For i.p. pre-exposure, 6-week-old mice were infected with 2.5×10^7 CFU SA. For SA challenge, mice were infected with 2.5×10^7 CFU SA, then spleen and kidneys were harvested 24h after infection, homogenized and diluted in phosphate-buffered saline (PBS), and plated on THB agar plates for CFU enumeration. For i.d. infections, mice were shaved and chemically depilated with Nair with Baby Oil under isoflurane anesthesia at least 24 h prior to infection with $5x10^7$ SA. Skin lesions were measured daily. Areas of infection were measured using FIJI ImageJ.

Bacteria

For all SA experiments, unless otherwise stated, the CA-MRSA LAC strain was used. Overnight SA broth cultures were subcultured 1:200 in Todd Hewitt broth (THB) and grown to an optical density (OD) of 0.7–0.8, then washed in phosphate-buffered saline (PBS) to a final dilution with OD 0.4. SA inoculum was confirmed by colony-forming unit (CFU) enumeration on THB agar plate. Heat-killed SA was prepared by incubating bacterial suspension for 45 min at 56C.

METHOD DETAILS

Cloning and protein expression

Recombinant protein-expressing E. coli BL21 strains for IsdB, ClfA, IsdA, MntC, FhuD2, and SdrE were described previously.[56] BL21 strains expressing ClfA, IsdA, MntC, and SdrE were gifts from Dr. David Underhill (Cedars-Sinai). For the remaining proteins: the synthesized gene insert for the EsxA/EsxB fusion protein (EsxAB) were purchased from GenScript. The LukE gene was amplified from LAC using primers:

Forward: LukE(5′-TAAGGCCTCTGTCGAAAATACTAATATTGAAAATATTGGT-3′) and.

Reverse: LukE(5′-CAGAATTCGCAAGCTTTAATTATGTCCTTTCACTTT-PRIMER-3′)

The EsxA gene was added to the N-terminus of EsxB genes with a Gly4Ser linker. The EsxAB synthesized gene insert and the LukE PCR product were then cloned into pET6xHN Expression Vector using the In-Fusion Cloning Kit (Clontech Laboratories). The nontoxigenic protein A, SpA_{KKAA} with four amino acid substitutions in each immunoglobulin binding domains (IgBD) was modified per previous study. For mutant protein A (SpA_{KKAA}), and alpha toxoid (Hla(H35L)), synthesized gene inserts cloned into pET15b expression vector were purchased from GenScript. All protein-expressing plasmids were transformed into E. coli BL21 (DE3) cells (New England Biolabs, NEB). To produce recombinant proteins, vector-containing E. coli BL21 were grown to OD0.6-0.7, then induced with 1mM of isopropyl-β-D-thiogalactoside (IPTG) for 3 h. Culture pellets were collected and suspended in lysis buffer (50 mM Tris-HCl (pH 8.0), 0.1 M NaCl, 2mM MgCl2, 10 mM imidazole, 0.1% Tween 80, 1% Triton X-100, PMSF, lysozyme (2 mg/ml). His-tagged proteins were purified from the clarified lysate by His60 Ni Superflow Resin (Takara) chromatography. The column was washed with 20mM Tris-HCl (pH 8.0), 150mM NaCl, 0.1% Tween 80 and His-tagged proteins were eluted with 300mM imidazole, 20 mM Tris-HCl (pH 7.5), 150 mM NaCl and 0.1% Tween-80. Purified proteins were concentrated using Amicon Ultra 50K centrifugal filter (Millipore). Endotoxin was removed from concentrated proteins using endotoxin removal spin columns (PierceTM). Accuracy of recombinant proteins were validated using protein sequencing by LC-MS (UCSD Biomolecular and Proteomics Mass Spectrometry Facility).

Antibody measurements (ELISA)

Total and antigen-specific antibody levels in human and mouse sera were measured by ELISA. Sera were serially diluted in PBS containing 1% BSA and added to 96-well high-binding plates coated with recombinant proteins (10 μg/ml). Bound antibodies were detected by horseradish peroxidase (HRP)-conjugated goat anti-mouse IgG or donkey anti-human IgG (BioLegend). Quantitative antibody measurements were performed using commercially purchased human and mouse IgG standards (ThermoFisher Scientific)

Antibody purification

Human sera were obtained from anonymized adult human volunteers. Mouse immune sera were generated by 3x i.p. SA infection with 3.5×10^7 CFU, or 3x immunization with 60μg IsdB adjuvanted with 600μg alum as previously described.[13] Total mouse IgG were purified from naive and immune mouse sera using Pierce Protein G Agarose (ThermoFisher Scientific), and total human IgG were purified from human sera using Pierce Protein A Agarose (ThermoFisher Scientific). Antigen-specific antibodies were purified from immune mouse or human sera using recombinant proteins immobilized in NHS-activated agarose columns (ThermoFisher Scientific). An example of the purity of human and mouse purified antibodies is shown in Figure S2B.

Antibody competition

Biotinylated purified serum antibody (anti-Hla or anti-ClfA) or commercially purchased monoclonal antibody (Suvratoxumab or Tefibazumab) were added (concentrations provided in respective figure legends) to 96-well high-binding plates coated with recombinant proteins. After incubation, unbound antibodies were washed with PBS containing 0.05% Tween 20. Competing antibodies were then added and incubated, and unbound antibodies were washed. Retained biotinylated antibodies were detected by horseradish peroxidase (HRP)-conjugated Avidin.

Immunization with vaccine proteins

Mice were immunized i.p. three times with optimized dose of vaccine protein (IsdA, EsxAB: 30μg/dose; MntC, SdrE, ClfA, SpA_{KKAA}, Hla_{H35L}, LukE, FhuD2: 60μg) plus aluminum hydroxide (alum, InvivoGen) (600μg per dose) or with aluminum hydroxide alone at 7-day intervals. Mouse sera were screened for reactivity to the vaccines by ELISA.

Adoptive transfer of serum or IgG

Immune sera were harvested 7 days after the final vaccination. IgG were purified from immune sera as described above. Sera and IgG were diluted to a final volume of 200μL and were injected i.v. into 8-week-old C57BL/6 mice 24 h prior to systemic i.p. SA challenge, or into 8-week-old CD1 mice 24 h prior to local skin SA challenge. Spleen and kidneys were harvested 24h after challenge, homogenized and diluted in PBS, then plated on THB agar plates for CFU enumeration.

Opsonophagocytic killing assay/SA survival assay

OPK assay was performed as previously described.[57] Mouse neutrophils were isolated from bone marrow by MojoSort Mouse Neutrophil Isolation Kit (BioLegend). Overnight culture of SA was subcultured 1:200 in THB and grown to OD 0.7–0.8. SA was washed

and resuspended in PBS. Prepared SA was incubated with mouse or human sera or purified antibodies at 37°C for 15 min, then added to 2x10^5 mouse neutrophils at a multiplicity of infection (MOI) of 1:0.1 in the presence of 2% normal mouse or 2.5% baby rabbit serum. Following incubation at 37°C for 1h with agitation at 200rpm, samples were serially diluted and plated on THB agar plates for CFU enumeration. OPK assay performed using mouse or baby rabbit serum produced similar results (Figure S2F; S3C). Compatibility of human IgG subclasses for mouse Fc gamma Receptors has been carefully studied and shown to have similar affinity of binding to mouse IgG subclass, which validated the use of the mouse neutrophils with human neutrophils.[58]

Culture of THP-1 cells

THP-1 cells were maintained in RPMI Medium 1640 (Gibco) supplemented with 10% fetal bovine serum, 5mL 1M HEPES, 5mL buffer, 50. Cells were incubated in 37C static incubation, supplemented with 5% CO2. At approximately 75% density, cells were passaged every 3 days, not exceeding 10 passages for experimental use. Prior to the experiment, cells were washed and resuspended in RPMI to a density of 2×10^6/mL.

Cytotoxicity/toxin neutralization assay

Hla-/LukED-mediated cytotoxicity assay is performed as previously described.[59] Corresponding purified human or mouse antitoxin antibodies were diluted in PBS in 96-well round-bottom plates and incubated with 0.25 μg/mL recombinant Hla (Sigma) or 0.375 μg/mL LukE/LukD (Mayflower Bioscience) for 15 min at 37°C, then 2x10^5 THP-1 monocytes were added. Following static incubation for 4 h at 37°C with 5% CO2, the remaining non-lysed cells were pelleted, and the supernatants were transferred into a new 96-well flat-bottom plate. Cytolysis-mediated LDH release was quantified using commercially purchased LDH detection kit (Takara).

Alpha toxin antibody avidity assay

Purified antibodies were diluted 1:1000 in PBS containing 1% BSA and added to 96-well high-binding plates coated with recombinant alpha toxin (10 μg/ml, Sigma). After incubation for 2hrs, the plates were treated with different concentration of urea (0–7 M) with 0.05% Tween 20 in PBS then washed. Bound antibodies were detected by horseradish peroxidase (HRP)-conjugated goat anti-mouse IgG or donkey anti-human IgG (BioLegend).

Adoptive transfer of sera or purified antibodies

Immune sera were generated by SA infection or immunization as described above. Human sera were obtained from anonymized adult human volunteers. Total mouse IgG were purified from mouse sera using Pierce Protein G Agarose (ThermoFisher Scientific), and total human IgG were purified from human sera using Pierce Protein A Agarose (ThermoFisher Scientific). Vaccine-specific antibodies were purified from mouse or human sera using vaccine proteins immobilized in NHS-activated agarose columns (ThermoFisher Scientific). Suvratoxumab, a monoclonal antibody against Hla used in clinical trials[60] was purchased from Creative Biolabs (TAB-463CQ). Immunized sera or purified antibodies were diluted in PBS and injected i.v. into recipient mice.

Flow cytometric analysis of antibody binding to *S. aureus*

For measurement of IsdB, ClfA and SdrE antibody binding to *S. aureus* cell surface, 1×10^7 CFU of LAC or SpA mutant strains were washed and incubated with 5 μg of antibody, then FITC-conjugated anti-mouse IgG. Fluorescence intensity was analyzed by FACSCanto (BD Biosciences) and FCS Express software (*De Novo* software).

SDS-PAGE and immunoblot

Purified antibodies or recombinant ClfA were denatured by adding 4x SDS loading dye (200 mM Tris-HCl pH 6.8, 8% SDS, 40% glycerol, 4% β-mercaptoethanol, 50 mM EDTA, 0.08% bromophenol blue) and boiling at 95°C for 5 min 10 μL of each sample was loaded into each well of NuPAGE 4 to 12% (ThermoFisher) and the proteins were separated by electrophoresis at 120 V for 2 h. Part of the gel was excised and stained with InstantBlue (expedeon). The other part was transferred to PVDF membrane (Bio-Rad) via wet transfer with XCell II Blot module at 100V for 2 h. Membranes were washed in TBST (25 mM Tris-HCL pH7.5, 300 mM NaCl, 0.05% Tween 20), blocked in 5% BSA/TBS for 1 h at RT, and incubated with depleted human serum or purified antibodies diluted 1:1000 in 5% BSA/TBST for 1 h at RT. After serum incubation, membranes were probed with HRP-conjugated anti-human IgG diluted 1: 20,000 in 5% BSA/TBST for 1 h at RT. The immunoreactive proteins were detected by Pierce ECL Western Blotting Substrate and chemiluminescent signals were captured by using a CCD camera (Fujifilm LAS-3000).

QUANTIFICATION AND STATISTICAL ANALYSIS

All statistical analyses were performed using GraphPad Prism version 7 for Windows, GraphPad Software, San Diego, CA, www.graphpad.com. Specific statistical analyses were noted in the figure legends. Two-group analysis used unpaired Student's *t* test (two-tailed tests). *In vivo* experiments were analyzed using non-parametric Mann-Whitney U-test. Comparisons of multiple groups were performed using one-way ANOVA, with Kruskal-Wallis test in the case of missing normality. Data were presented as mean ± standard deviation (SD), unless otherwise indicated. Statistical significance was assigned as ***$p \leq 0.001$; **$p \leq 0.01$; *$p \leq 0.05$; $p > 0.05$: ns (not significant).

Cell Reports Medicine, Volume *5*

Supplemental information

The characteristics of pre-existing humoral imprint determine efficacy of *S. aureus* vaccines and support alternative vaccine approaches

J.R. Caldera, Chih-Ming Tsai, Desmond Trieu, Cesia Gonzalez, Irshad A. Hajam, Xin Du, Brian Lin, and George Y. Liu

Cell Reports Medicine, Volume 5

Supplemental information

The characteristics of pre-existing humoral imprint determine efficacy of *S. aureus* vaccines and support alternative vaccine approaches

JR Caldera, Chih-Ming Tsai, Desmond Trieu, Cesia Gonzalez, Irshad A. Hajam, Xin Du, Brian Lin, George Y. Liu

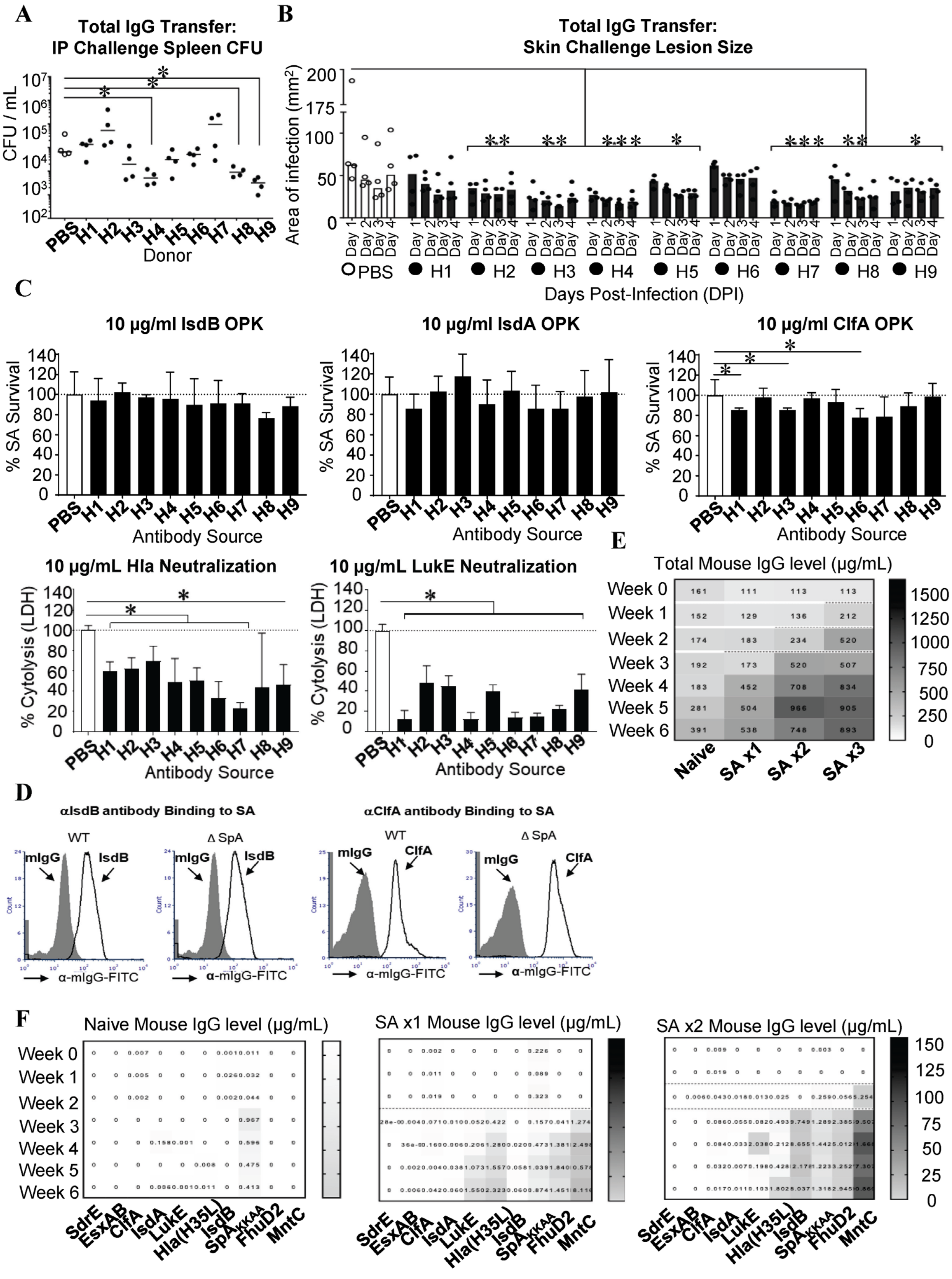
A
Total IgG Transfer: IP Challenge Spleen CFU
CFU / mL
Donor
PBS H1 H2 H3 H4 H5 H6 H7 H8 H9
B
Total IgG Transfer: Skin Challenge Lesion Size
Area of infection (mm²)
Days Post-Infection (DPI)
PBS H1 H2 H3 H4 H5 H6 H7 H8 H9
C
10 μg/ml IsdB OPK
10 μg/ml IsdA OPK
10 μg/ml ClfA OPK
% SA Survival
Antibody Source
10 μg/mL Hla Neutralization
10 μg/mL LukE Neutralization
% Cytolysis (LDH)
E
Total Mouse IgG level (μg/mL)
Week 0
Week 1
Week 2
Week 3
Week 4
Week 5
Week 6
Naive
SA x1
SA x2
SA x3
D
αIsdB antibody Binding to SA
αClfA antibody Binding to SA
WT
Δ SpA
mIgG
IsdB
ClfA
α-mIgG-FITC
F
Naive Mouse IgG level (μg/mL)
SA x1 Mouse IgG level (μg/mL)
SA x2 Mouse IgG level (μg/mL)
SdrE EsxAB ClfA IsdA LukE Hla(H35L) IsdB SpA KKAA FhuD2 MntC

Figure S1 Quantitative and functional assessments of human and mouse anti-SA humoral imprints, related to Figure 1.

(A) Post-challenge bacterial burden in kidneys of naïve C57BL/6 mice adoptively transferred total purified human IgG, or PBS, then challenged i.p. with SA.

(B) Skin lesion size after adoptive transfer of total human IgG or PBS, into naïve mice challenged i.d., measured daily for 4 days. Each point represents an individual mouse. Bar corresponds to the median.

(C) *In vitro* assessment of relative antigen-specific antibody function by OPK, and toxin-neutralization. Results are normalized to their respective control using normal mIgG.

(D) Validation of bacterial expression of IsdB/ClfA and binding of purified IsdB- andClfA- specific antibodies to log-phase growth of wild type SA LAC and isogenic SpA-mutant analyzed by flow cytometry. The gray histogram represents staining with FITC-conjugated anti-mouse IgG only; the bolded histogram line represents staining with IsdB antibodies, then secondary antibody.

(E) Total IgG in serum of naïve, 1x, and 2x SA-exposed mice, over 7 weeks. Dashed line denotes timepoints of SA exposure. Values in each cell correspond to the median titer from n=5 mice.

(F) Antigen-specific IgG corresponding to 10 proposed vaccine candidates, over 7 weeks from naïve and SA x1 and x2 infected mice. Dashed line denotes time of SA exposure. Values in each cell correspond to the median titer from n=5 mice. Ranking of antigens is based on average titer among the timepoints.

Unless otherwise stated, C57BL/6 mice were used. Each point represents an individual mouse (A and B); line or bar corresponds to the median (A and B). Bar represents group median; error bars represent means ± SD (C). n.s., not significant, $*p < 0.05$, $**p < 0.01$, and $***p < 0.001$. One-way ANOVA (A and C), Two-way ANOVA with Bonferroni correction (B).

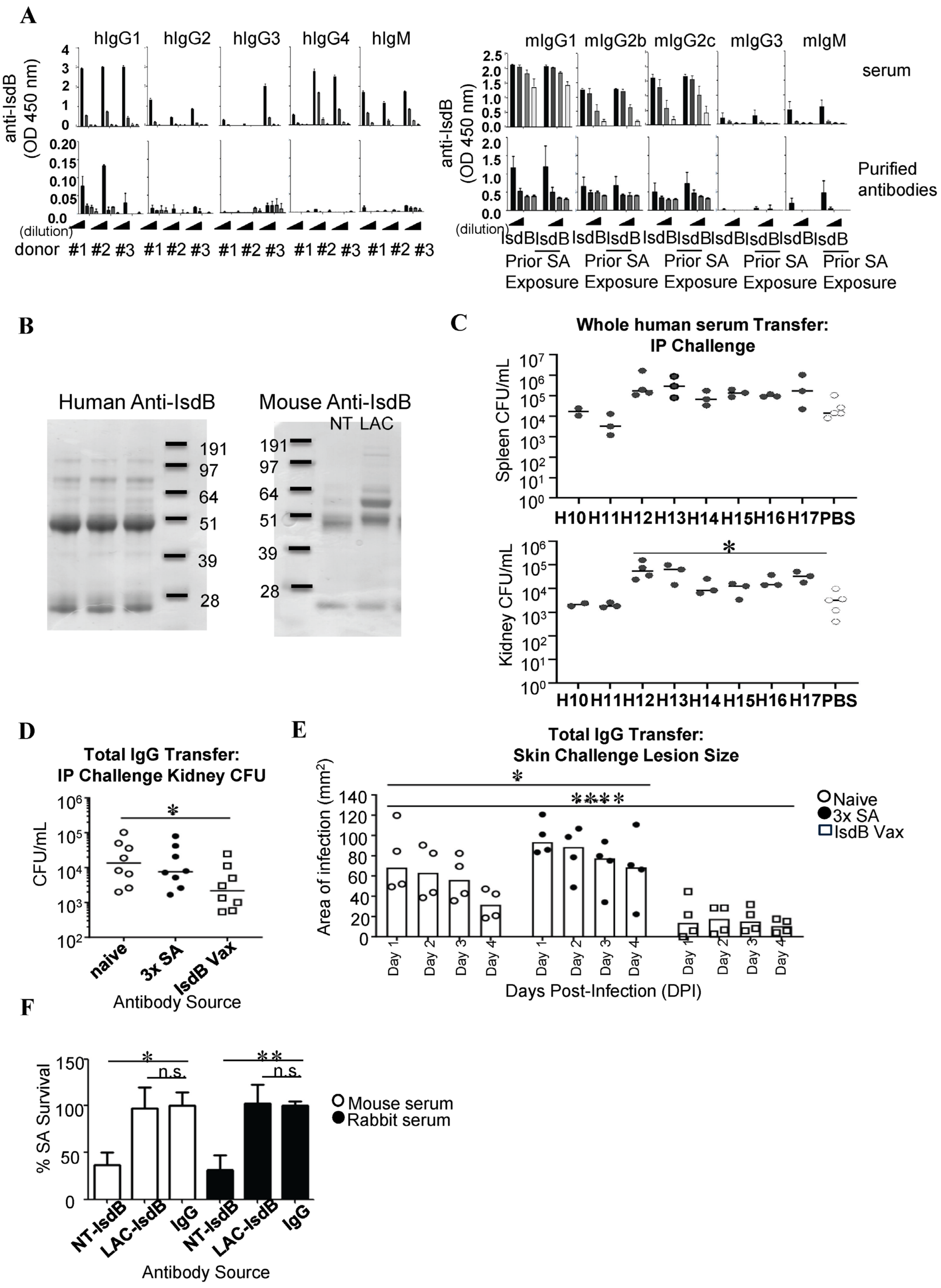
A
hIgG1 hIgG2 hIgG3 hIgG4 hIgM
anti-IsdB (OD 450 nm)
(dilution)
donor #1 #2 #3 #1 #2 #3 #1 #2 #3 #1 #2 #3 #1 #2 #3
mIgG1 mIgG2b mIgG2c mIgG3 mIgM
serum
Purified antibodies
IsdB IsdB Prior SA Exposure
B
Human Anti-IsdB
Mouse Anti-IsdB
NT LAC
191 97 64 51 39 28
C
Whole human serum Transfer: IP Challenge
Spleen CFU/mL
Kidney CFU/mL
H10 H11 H12 H13 H14 H15 H16 H17 PBS
*
D
Total IgG Transfer: IP Challenge Kidney CFU
CFU/mL
naive 3x SA IsdB Vax
Antibody Source
E
Total IgG Transfer: Skin Challenge Lesion Size
Area of infection (mm²)
Naive
3x SA
IsdB Vax
Day 1 Day 2 Day 3 Day 4
Days Post-Infection (DPI)
*
****
F
% SA Survival
n.s.
Mouse serum
Rabbit serum
NT-IsdB LAC-IsdB IgG NT-IsdB LAC-IsdB IgG
Antibody Source

Figure S2 Quantitative and functional assessments of human and mouse anti-SA humoral imprints, related to Figure 1.

(A) Isotype profiling of anti-IsdB antibodies from human (n=3) or mouse (n=5) whole or column-purified serum samples. IsdB-vaccinated naïve and SA-experienced mice were the sources of murine sera.

(B) SDS-PAGE analysis of representative purified antibodies: IsdB-specific antibodies purified from healthy human serum (n=3) and IsdB-vaccinated previously non-treated (NT) and IsdB-vaccinated SA-experienced mice (SA) (n=5). Please note that the 57KD band from the SA sample is 70% albumin,2% IgG and 9.93% IgM based on MS analysis.

(**C**) Post-i.p challenge SA burden in kidneys of mice adoptively transferred of 100μl human sera from subjects H10-H17 i.p.

(D) Post-challenge bacterial burden in kidneys of naïve C57BL/6 mice adoptively transferred total purified mouse IgG, then challenged i.p. with SA. Bar corresponds to the median.

(E) Skin lesion size after adoptive transfer of total mouse IgG, into naïve mice challenged i.d., measured daily for 4 days.

(F) *In vitro* comparison of relative protective serum anti-IsdB function by OPK with supplementation of mouse (left) or baby rabbit serum (right). Results are normalized to mIgG control.

Unless otherwise stated, C57BL/6 mice were used. Each point represents an individual mouse (C-E); line or bar corresponds to the median (C-E). Bar represents group median; error bars represent means ± SD (A and F). n.s., not significant, $*p < 0.05$, $**p < 0.01$, and $***p < 0.001$. One-way ANOVA (D and F), Two-way ANOVA with Bonferroni correction (E).

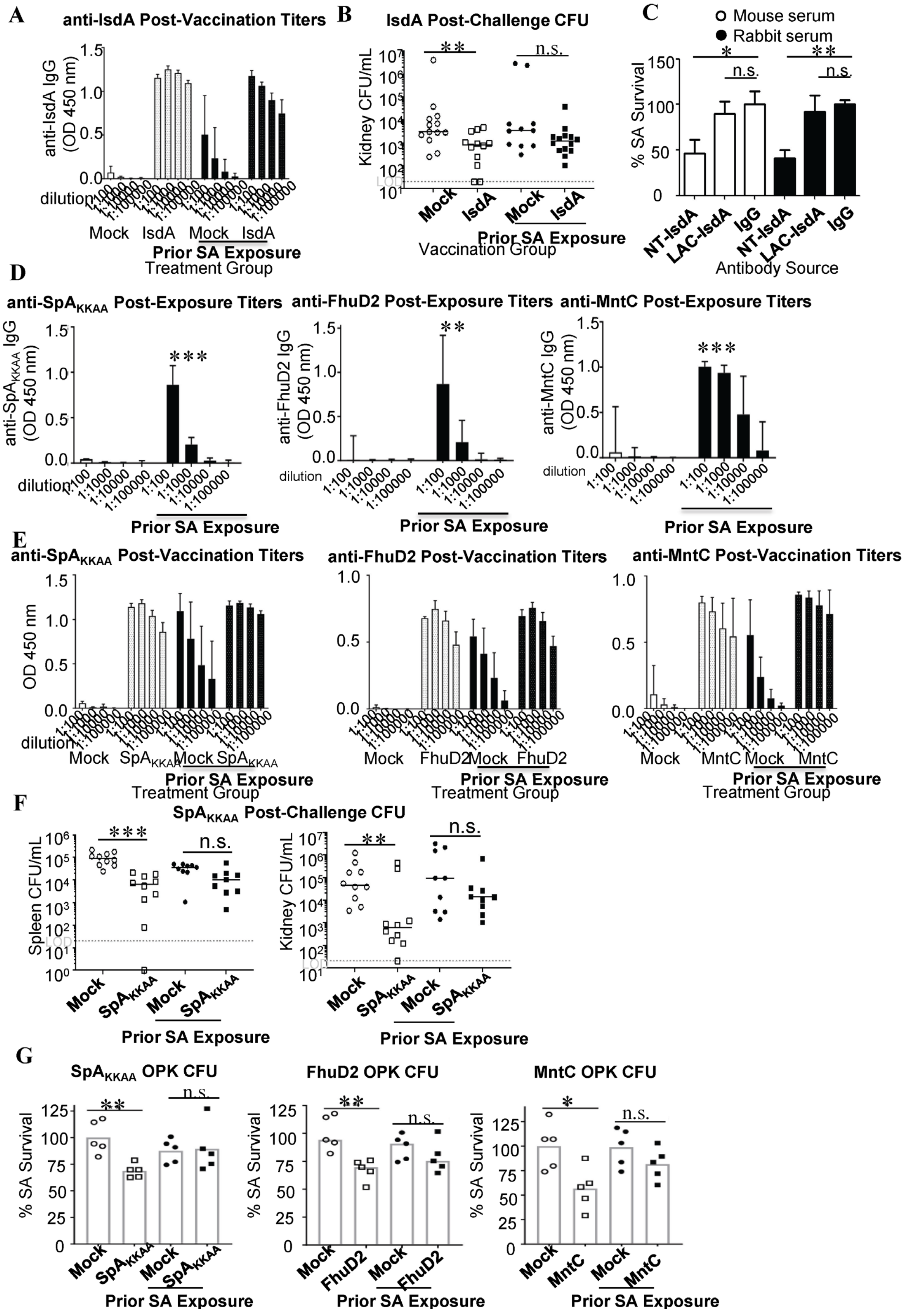
A
anti-IsdA Post-Vaccination Titers
B
IsdA Post-Challenge CFU
C
Mouse serum
Rabbit serum
NT-IsdA
LAC-IsdA
IgG
Antibody Source
Mock
IsdA
Prior SA Exposure
Treatment Group
Vaccination Group
D
anti-SpA$_{KKAA}$ Post-Exposure Titers
anti-FhuD2 Post-Exposure Titers
anti-MntC Post-Exposure Titers
E
anti-SpA$_{KKAA}$ Post-Vaccination Titers
anti-FhuD2 Post-Vaccination Titers
anti-MntC Post-Vaccination Titers
F
SpA$_{KKAA}$ Post-Challenge CFU
Spleen CFU/mL
Kidney CFU/mL
G
SpA$_{KKAA}$ OPK CFU
FhuD2 OPK CFU
MntC OPK CFU
% SA Survival

Figure S3 Active immunizations targeting CWA are not protective in SA-experiencd mice, related to Figure 2.

(A) CWA-specific titers from n=5 vaccinated naïve and SA-experienced mice, 7 days after the last vaccination.

(B) Post-challenge bacterial burden in kidneys of mock- or IsdA-immunized naïve or SA-experienced mice.

(C) *In vitro* assessment of relative protective serum anti-IsdA function by OPK with serum supplement from mouse serum (open bar) or baby rabbit serum (black bar). Results are normalized to mIgG control.

(D-E) CWA-specific titers from n=10 naïve and SA-experienced mice, 7 days after the last SA exposure (D), and 7 days after last immunization (E).

(F) Post-challenge bacterial burden in kidneys of mock- or SpA_{KKAA}- immunized naïve or SA-experienced mice.

(G) *In vitro* assessment of relative protective serum function by OPK. Results are normalized to their respective control. Bar corresponds to the median. Each point represents an individual mouse. Bar corresponds to the median. Error bar corresponds to the range.

Unless otherwise stated, C57BL/6 mice were used. Bar represents group median; error bars represent means ± SD (A and C-E). Each point represents an individual mouse (B and F); bar corresponds to the median and dashed lines indicate the limit of detection (LOD) (B and F). $*p < 0.05$, $**p < 0.01$, and $***p < 0.001$. One-way ANOVA (B-C, and F-G).

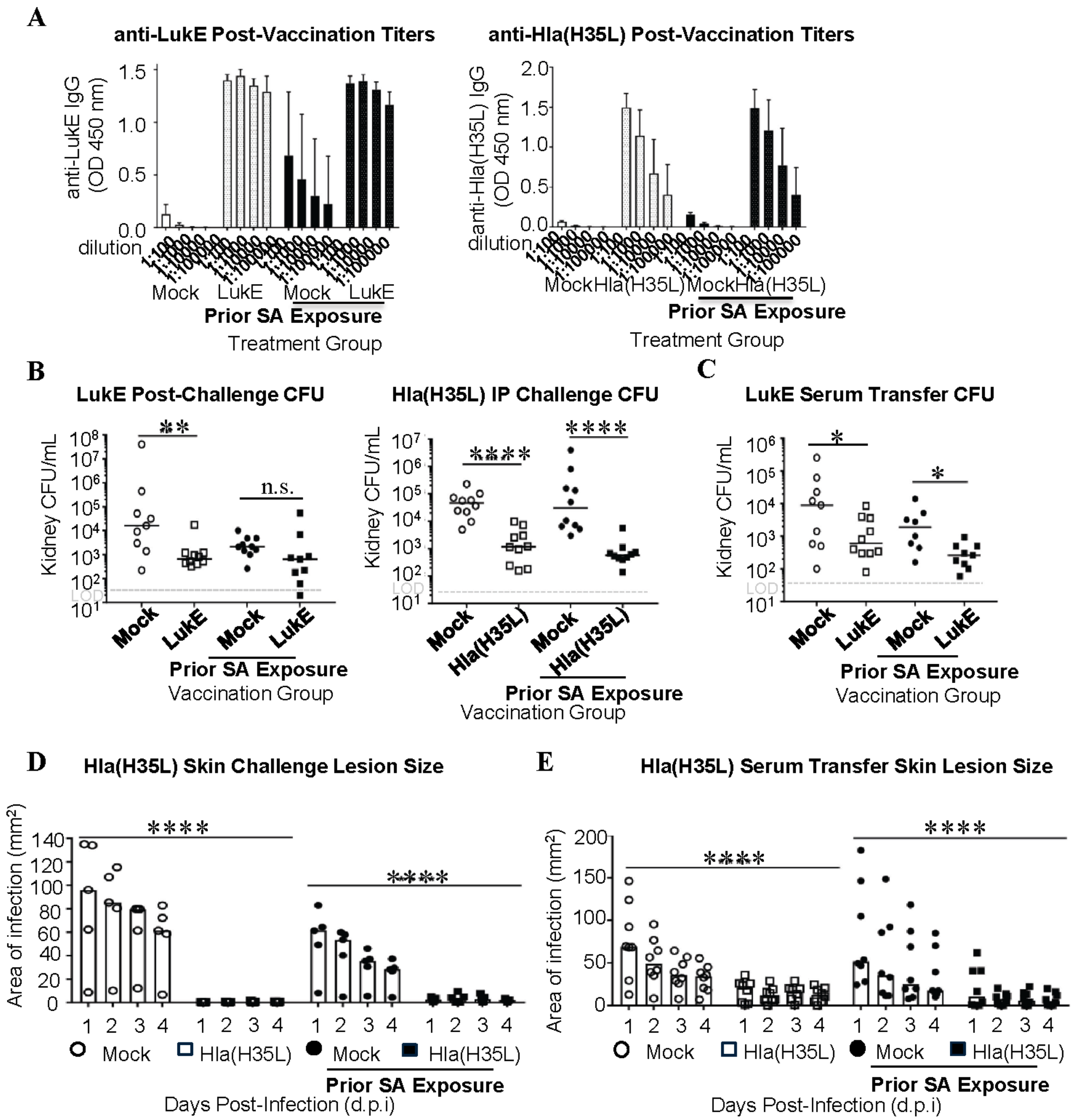

Figure S4 Active immunizations targeting toxin antigens are protective in SA-experienced mice, related to Figure 2.

(A) Toxin-specific titers from n=5 vaccinated naïve and SA-experienced mice, 7 days after the last vaccination. Bar corresponds to the median. Error bar corresponds to the range.

(B) Post-i.p. challenge bacterial burden in kidneys of mock-, LukE- or $Hla_{(H35L)}$-vaccinated naïve or SA-experienced mice. Each point represents an individual mouse. Line corresponds to the median.

(C) Post-i.p. challenge bacterial burden in kidneys of mice adoptively transferred serum from mock-, or LukE-vaccinated naïve or SA-experienced mice. Each point represents an individual mouse. Line corresponds to the median.

(D) Skin lesion size in mock- or $Hla_{(H35L)}$-vaccinated CD-1 naïve or SA-experienced mice measured daily for 4 days. Each point represents an individual mouse. Bar corresponds to the median. Dashed line corresponds to skin lesion size at 2d.p.i. of PBS control.

(E) Skin lesion size of CD-1 mice adoptively transferred serum from mock- or $Hla_{(H35L)}$-vaccinated mice measured daily for 4 days.

Unless otherwise stated, C57BL/6 mice were used. Bar represents group median. Each point represents an individual mouse (B-E); bar corresponds to the median and dashed lines indicate the limit of detection (LOD) (B and C). $*p < 0.05$, $**p < 0.01$, and $***p < 0.001$. One-way ANOVA (B and C) or two-way ANOVA with Bonferroni correction (D and E).

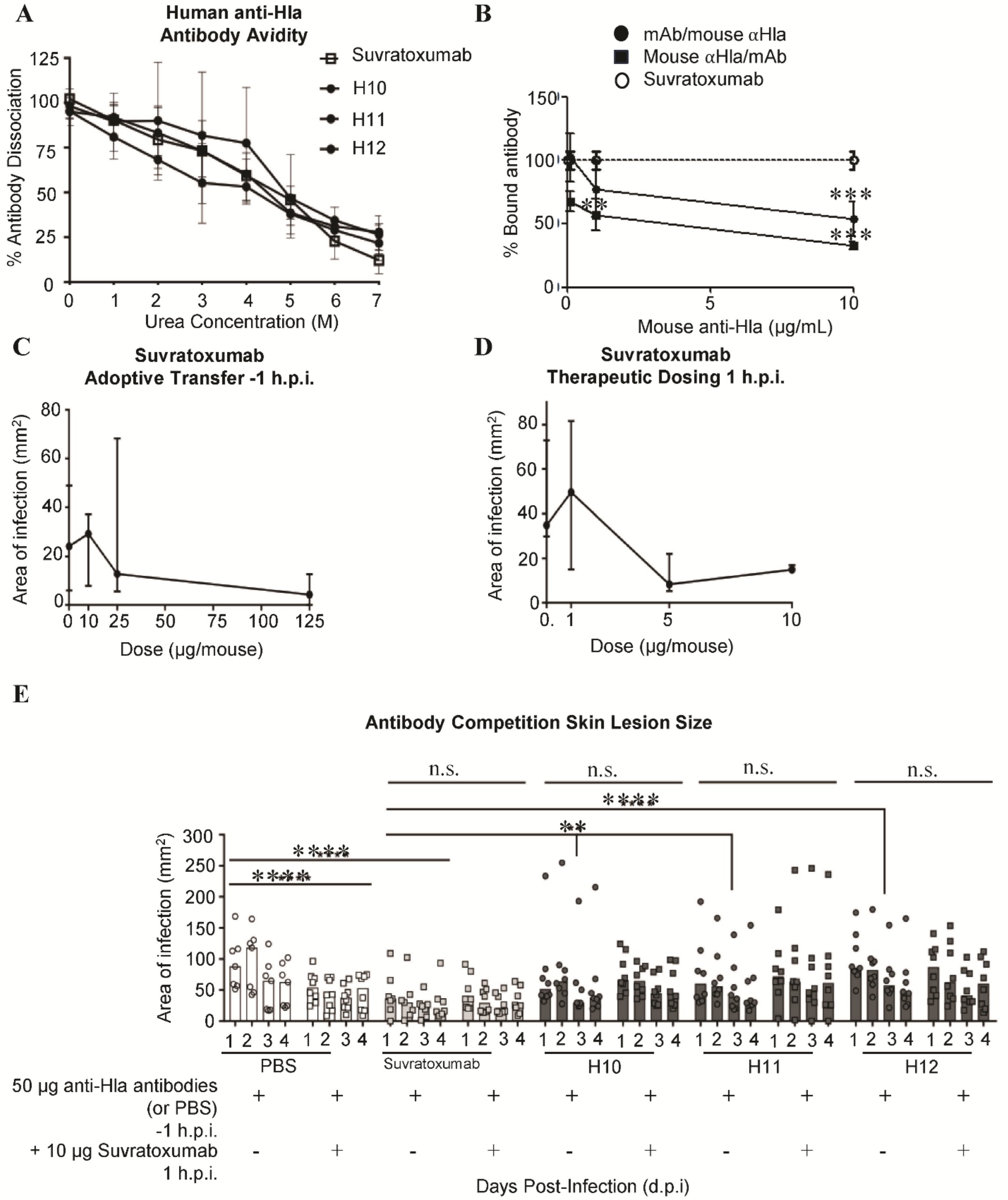

Figure S5 Pre-existing human anti-Hla antibodies blunt the efficacy of anti-Hla monoclonal antibody Suvratoxumab, related to Figure 3.

(A) Retention of anti-Hla antibodies (1µg/mL) with 0M-7M urea treatment. Each point represents 3 technical replicates. Error bar corresponds to range.

(B) Binding competition between purified mouse anti-Hla and Suvratoxumab, in an ELISA plate assay. Binding of recombinant Hla(H35L) by Suvratoxumab (1 ug/mL) or Hla-specific antibodies purified, from mice that have been SA-infected 3x (0.1-10 ug/ml). Suvratoxumab/mouse αHla: Suvratoxumab first, then mouse αHla; Mouse αHla/Suvratoxumab: mouse □Hla first, then Suvratoxumab; Suvratoxumab: Suvratoxumab only. Each point represents 3 technical replicates.

(C) Skin lesion size in CD-1 mice (n=3) that were adoptively transferred PBS, 10µg, 25µg, or 125µg Suvratoxumab, and then infected i.d. one hour later with SA. Skin lesions are measured 2 d.p.i.

(D) Skin lesion size of CD-1 mice after therapeutic administration of Suvratoxumab (10µg/mouse) or PBS 1h.p.i., measured daily for 4 days. Each point represents an individual mouse. Bar corresponds to the median. Dashed line corresponds to skin lesion size at 2d.p.i. of PBS control.

(E) Skin lesion size 1-4 d.p.i. in CD-1 mice passively immunized with anti-Hla antibodies (50µg/mouse), or PBS 1 hour prior to infection, followed by Suvratoxumab treatment (10µg/mouse) or PBS 1-hour post-infection. Each point represents an individual mouse.

Bar corresponds to the median. Error bars represent mean ± SD. $*p < 0.05$, $**p < 0.01$, and $***p < 0.001$. Student's t test (A, B) or two-way ANOVA with Bonferroni correction (E).

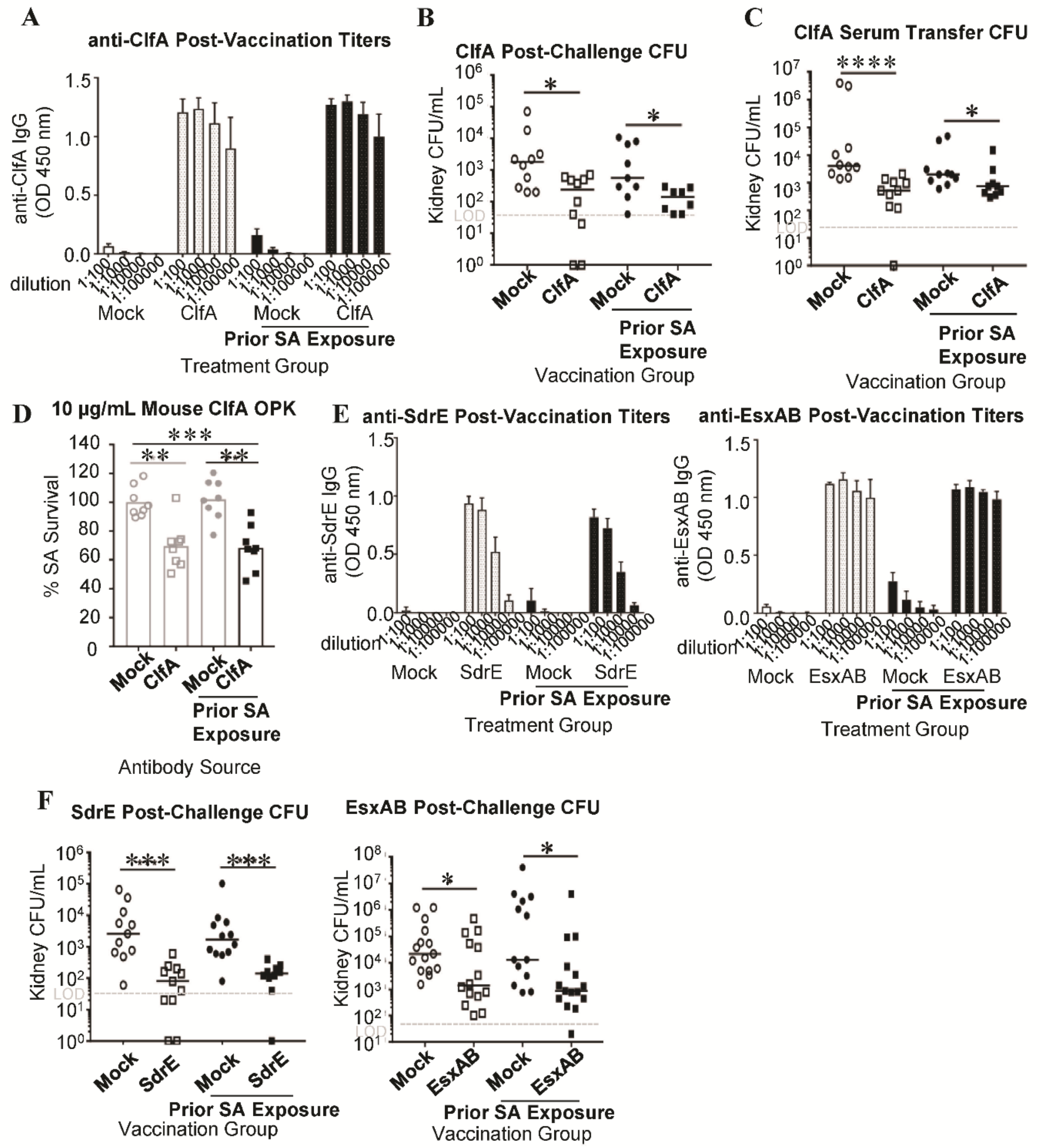

Figure S6 Subdominant SA CWA induce protective immunity in SA-experienced mice, related to Figure 4.

(A and E) ClfA-, SdrE- and EsxAB-specific titers from n=5 vaccinated naïve and SA-experienced mice, 7 days after the last SA vaccination. Bar corresponds to the median. Error bar corresponds to the range.

(B and F) Post-challenge bacterial burden in kidneys of mock- or (ClfA-, EsxAB- and SdrE-) vaccinated naïve or SA-experienced mice. Bar corresponds to the median.

(C) Post-challenge bacterial burden in kidneys of mice adoptively transferred serum from mock- or ClfA-vaccinated naïve or SA-experienced mice.

(D) *In vitro* assessment of relative ClfA-specific antibody function by OPK with purified anti-ClfA antibodies. Results are normalized to mIgG control. Bar corresponds to the mean of 8 biological *replicates from two independent experiments*.

C57BL/6 mice were used. Bar represents group median; error bars represent mean ± SD (A, E). Each point represents an individual mouse (B, C and F); bar corresponds to the median and dashed lines indicate the limit of detection (LOD) (B-C and F). $*p < 0.05$, $**p < 0.01$, and $***p < 0.001$.$*p < 0.05$, $**p < 0.01$, and $***p < 0.001$. one-way ANOVA (B-D and F).
